# Optimal timing of allogeneic hematopoietic stem cell transplantation in patients with myelodysplastic syndrome

**DOI:** 10.1002/ajh.23458

**Published:** 2013-04-20

**Authors:** Emilio Paolo Alessandrino, Matteo G Della Porta, Luca Malcovati, Christopher H Jackson, Cristiana Pascutto, Andrea Bacigalupo, Maria Teresa van Lint, Michele Falda, Massimo Bernardi, Francesco Onida, Stefano Guidi, Anna Paola Iori, Raffaella Cerretti, Paola Marenco, Pietro Pioltelli, Emanuele Angelucci, Rosi Oneto, Francesco Ripamonti, Alessandro Rambaldi, Alberto Bosi, Mario Cazzola, on behalf of Gruppo Italiano Trapianto di Midollo Osseo (GITMO)

**Affiliations:** 1 Department of Hematology Oncology, Fondazione IRCCS Policlinico San MatteoPavia, Italy; 2 Department of Molecular Medicine, University of PaviaPavia, Italy; 3 MRC Biostatistics Unit, Institute of Public HealthCambridge, United Kingdom; 4 Department of Hematology, San Martino HospitalGenova, Italy; 5 Department of Hematology, San Giovanni Battista HospitalTurin, Italy; 6 Department of Oncology, Hematology and BMT Unit, San Raffaele Scientific InstituteMilan, Italy; 7 Hematology and Bone Marrow Transplantation Center, Fondazione IRCCS Ospedale Maggiore Policlinico, University of MilanMilan, Italy; 8 Division of Hematology and Bone Marrow Transplantation, Azienda Ospedaliera Universitaria CareggiFlorence, Italy; 9 Department of Cellular Biotechnologies and Hematology, Sapienza UniversityRome; 10 Department of Hematology, Rome Transplant Network, Stem Cell Transplant UnitPoliclinico Tor Vergata, Rome; 11 Department of Hematology Oncology, Ospedale Niguarda Ca' GrandaMilan, Italy; 12 Division of Hematology and Transplant Unit, Ospedale San GerardoMonza, Italy; 13 Hematology and Bone Marrow Transplantation Unit, Ospedale Oncologico di Riferimento Regionale Armando BusincoCagliari, Italy; 14 Division of Hematology, Ospedali RiunitiBergamo, Italy

## Abstract

Allogeneic hematopoietic stem cell transplantation (HSCT) represents the only curative treatment for patients with myelodysplastic syndrome (MDS), but involves non-negligible morbidity and mortality. Registry studies have shown that advanced disease stage at transplantation is associated with inferior overall survival. To define the optimal timing of allogeneic HSCT, we carried out a decision analysis by studying 660 patients who received best supportive care and 449 subjects who underwent transplantation. Risk assessment was based on both the International Prognostic Scoring System (IPSS) and the World Health Organization classification-based Prognostic Scoring System (WPSS). We used a continuous-time multistate Markov model to describe the natural history of disease and evaluate the effect of allogeneic HSCT on survival. This model estimated life expectancy from diagnosis according to treatment policy at different risk stages. Relative to supportive care, estimated life expectancy increased when transplantation was delayed from the initial stages until progression to intermediate-1 IPSS-risk or to intermediate WPSS-risk stage, and then decreased for higher risks. Modeling decision analysis on WPSS versus IPSS allowed better estimation of the optimal timing of transplantation. These observations indicate that allogeneic HSCT offers optimal survival benefits when the procedure is performed before MDS patients progress to advanced disease stages. Am. J. Hematol. 88:581–588, 2013. © 2013 Wiley Periodicals, Inc.

## Introduction

Myelodysplastic syndromes (MDS) are myeloid neoplasms that present as refractory cytopenia and typically occur in elderly people 1. Because of population aging, MDS represent one of the most common hematologic malignancies in Western countries nowadays, their annual incidence exceeding 20 per 100,000 persons over the age of 60 years 1.

MDS are highly heterogeneous from a clinical point of view, ranging from conditions with a near-normal life expectancy to forms approaching acute myeloid leukemia (AML) 2. This clinical heterogeneity reflects different somatic mutations responsible for clonal proliferation of myelodysplastic stem cells 3–5. In particular, spliceosome mutations, implying abnormalities of mRNA splicing in the pathogenesis of MDS, have variable prognostic relevance, depending on the mutated splicing factor 6–9.

From a practical point of view, the definition of individual risk requires the use of prognostic models. In 1997, Greenberg et al. developed the International Prognostic Scoring System (IPSS) 10, which has rapidly become a benchmark for clinical decision-making, clinical trials, and drug approval. Despite its usefulness, this scoring system has weaknesses 11, and has been recently revised with the development of the IPSS-R 12. Following the introduction of the World Health Organization (WHO) classification of MDS 13, a WHO classification-based Prognostic Scoring System (WPSS) was defined, based on WHO categories, cytogenetic abnormalities, and transfusion-dependency 14,15. WPSS proved to be able to provide dynamic prognostic information at any time of the disease course, and to predict the outcome of allogeneic hematopoietic stem cell transplantation (HSCT) 16.

Despite recent therapeutic progress, the only curative treatment for MDS patients remains allogeneic HSCT, which is considered as a conventional therapeutic option until the age of 65 in eligible patients. Its efficacy, however, is considerably limited by morbidity and mortality, resulting in a long-term survival rate of about 30% 17. Several issues must be taken into account when considering allogeneic HSCT and evaluating its benefits in the individual patient with MDS, and a crucial question is timing of transplantation 18. Considering the clinical course of MDS, the optimal timing of allogeneic HSCT would be a disease stage that provides the best overall life expectancy, accounting for both pretransplantation and posttransplantation survival. In fact, patients at early stages, especially those with a somatic mutation of *SF3B1*, may experience long periods with stable disease after diagnosis 2,6, and the risks of morbidity and mortality related to allogeneic HSCT would be unacceptably high for many of them.

However, a number of studies have shown that advanced disease stage at transplantation is associated with inferior overall survival 16,17. In particular, a recent study of the European Group for Blood and Marrow Transplantation clearly showed that advanced disease stage at transplantation was the major independent variable associated with an inferior 4-year overall survival in MDS patients 50 years or older 17. However, a previous decision analysis by the International Bone Marrow Transplant Registry (IBMTR) concluded that, whereas immediate transplantation was associated with maximal life expectancy in patients with intermediate-2- and high IPSS risk, for those with low and intermediate-1 IPSS-risk delayed transplantation offered optimal survival benefit 19. It was therefore concluded that the optimal timing of transplantation was at the time of disease progression from lower to higher IPSS risk groups. This study has substantially influenced clinical practice despite a number of intrinsic limitations 11. In particular, the IBMTR analysis considered patients at the time of MDS diagnosis, ignoring changes in their disease status that frequently occur before transplantation or leukemic evolution.

To overcome the above limitations, we carried out an ad hoc decision analysis in MDS patients aged up to 65 years, classified according to WHO criteria and stratified according to either the IPSS or WPSS. We used a continuous-time multistate Markov model to describe the natural history of disease and evaluate the effect of allogeneic HSCT on survival at different stages of disease.

## Methods

### Patients and study design

These investigations were approved by the Ethics Committee of the Fondazione Istituto di Ricovero e Cura a Carattere Scientifico (IRCCS) Policlinico San Matteo, Pavia, Italy. All procedures were carried out in accordance with the ethical standards of the Declaration of Helsinki.

We analyzed two cohorts comprising 1137 MDS patients. The first cohort (Pavia cohort) included 660 patients diagnosed with MDS according to WHO criteria 13,20 at the Fondazione IRCCS Policlinico San Matteo, Pavia, Italy, between 1992 and 2009. The second cohort (GITMO cohort) included 477 undergoing allogeneic HSCT for primary MDS or AML evolved from MDS between 1997 and 2009, and reported to the GITMO registry (Table I). Secondary AML was included in this analysis since this condition is very close to RAEB-2, and not infrequently difficult to be distinguished from this latter condition. Most of these patients with AML evolved from MDS had the condition previously defined as refractory anemia with excess of blasts in transformation (RAEB-t), characterized by 20–29% blasts in the bone marrow 21. Different conditioning regimens and different donor types had been employed as shown in Supporting Information Table I.

**TABLE I:** Clinical Characteristics of MDS Patients Belonging to the Pavia Cohort, Who Received Best Supportive Care, and to the GITMO Cohort, Who Had Received Allogeneic HSCT

		GITMO cohort	
		
Clinical characteristics	Pavia cohort	MDS	AML evolving from MDS^a^
Number of patients	660	337	140
Age (completed years; median, range)	63 (11–92)	48 (13–69)	46 (15–69)
Sex (male/female)	397 (60%)/263 (40%)	176 (52%)/161 (48%)	72 (51%)/68 (49%)
WHO classification^b^			
RCUD	105 (16%)	23 (7%)	
RARS	76 (12%)	11 (3%)	–
MDS del (5q)	42 (6%)	4 (1%)	
RCMD	234 (35%)	74 (22%)	
RAEB-1	93 (14%)	77 (23%)	
RAEB-2	110 (17%)	148 (44%)	
Hemoglobin (g/dL; median, range)	9.8 (3.8–16)	8.8 (7–12.4)	9 (6.8–11)
Absolute neutrophil count (×10^9^/L; median, range)	1.92 (0.58–19.00)	1.16 (0.1–11.5)	1.4 (0.2–9.4)
Platelet count (×10^9^/L; median, range)	125 (8–1420)	48 (3–491)	58 (2–319)
IPSS risk			
Low	222 (34%)	23 (7%)	
Intermediate-1	273 (41%)	118 (35%)	
Intermediate-2	127 (19%)	139 (41%)	
High	38 (6%)	57 (17%)	
WPSS risk			
Very-low	142 (21%)	–	
Low	183 (28%)	34 (10%)	
Intermediate	117 (18%)	74 (22%)	
High	176 (27%)	185 (55%)	
Very high	42 (6%)	44 (13%)	

In the Pavia cohort, all clinical variables were analyzed at the time of diagnosis. In the GITMO cohort, clinical variables were analyzed at the time of transplantation in patients undergoing upfront allogeneic HSCT, and at the time of remission-induction chemotherapy in those receiving treatment before transplantation.

a Including patients affected with RAEB in transformation according to the FAB classification.

b RCUD, refractory cytopenia with unilineage dysplasia; RARS, refractory anemia with ring sideroblasts; MDS del(5q), myelodysplastic syndrome with del(5q); RCMD, refractory cytopenia with multilineage dysplasia; RAEB-1, refractory anemia with excess blasts type 1; RAEB-2, refractory anemia with excess blasts type 2.

Disease-related risk was evaluated by using both the IPSS 10 and WPSS 14. With respect to this latter, we used the original prognostic model that included transfusion dependency, as this parameter has proved to have relevant prognostic significance for the outcome of allogeneic HSCT 22.

In the Pavia cohort, patients were essentially treated with best supportive care and regularly followed-up, and this allowed clinical data and disease staging to be monitored longitudinally. In the GITMO cohort, all clinical variables were analyzed at the time of transplantation in patients undergoing allogeneic HSCT upfront, and at the time of remission-induction chemotherapy in those receiving treatment before allogeneic HSCT (Table I, and Supporting Information Table I).

### Decision strategy

We adopted a continuous-time, multistate Markov approach to model the course of the disease in MDS patients and to assess the effect of allogeneic HSCT on overall survival 23,24. A multistate model describes how an individual moves between a series of states in time. Markov models are multistate models based on Markov processes, that is, stochastic processes with the property that the probability of moving to a particular state in the future only depends on this state and not on past states. Further information on Markov models is reported in Supporting Information Methods.

**Figure 1:**
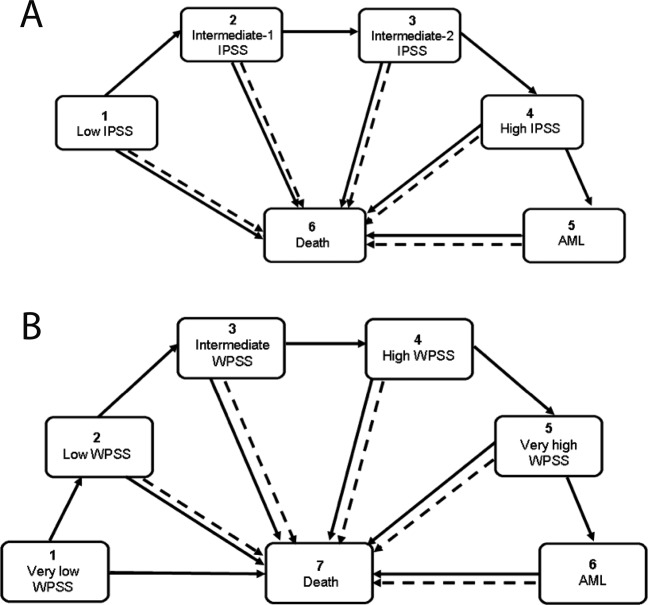
Markov continuous-time multistate models of the natural history of MDS. IPSS (A) and WPSS (B) risk scores were adopted as time-dependent indicators of the natural course of MDS. Allogeneic HSCT was modeled as a time-dependent covariate, and its effect on survival was estimated as a HR with respect to the “no allogeneic HSCT” category. Solid arrows represent transitions according to the natural course of the disease, while the effect of allogeneic HSCT on transitions is represented in each state by a dashed arrow.

A continuous-time Markov model was used to estimate the risk of progression from each disease state to the next one. We fitted two models based on IPSS and WPSS risk, respectively (Fig. 1). Each risk category was represented by a state in the model, and death was considered as absorbing state, that is, a state in which transitions to other states are not allowed. Transitions were allowed from any IPSS or WPSS risk state to the next one, to AML and to death. Transition intensity, that is, the instantaneous risk of moving to another state, was then estimated for each possible transition between states.

Allogeneic HSCT was modeled as a categorical time-dependent covariate, to allow for excess of mortality due to transplant-related causes. The effect of allogeneic HSCT on mortality in each disease state was estimated as a hazard ratio (HR) with respect to the “no allogeneic HSCT” category. Since allogeneic HSCT does not represent an option for very low WPSS risk patients, the HRs for transplantation in this state were not modeled. A more detailed technical description of the models employed is reported in Supporting Information Methods and Supporting Information Fig. 1.

The expected survival, that is, the expected time spent by a patient in the model before reaching the absorbing state (death), under different transplant policies was calculated algebraically for the fitted Markov model. These calculations were validated by microsimulation, and a confidence interval was obtained by bootstrap resampling.

Life expectancy was also estimated accounting for quality of life (QoL), based on quality-adjusted life years (QALY). We made QoL adjustments by incorporating utilities into estimation of life expectancy. Utilities are numerical representations of the perceived value of a given health state and are expressed as values between 0 (a health state equivalent to being dead) and 1 (perfect health) 25. To account for worsening of QoL with disease progression or transplant-related complications, we defined plausible utilities using previously published data 19,25–27. With respect to the natural course of the disease, we assigned QALY = 1 to the very low WPSS risk; QALY = 0.95 to low and intermediate WPSS, or low and intermediate-1 IPSS; and QALY = 0.90 to high and very high WPSS, or intermediate-2 and high IPSS. Evolution to AML was assigned QALY = 0.85. In patients receiving transplantation, the onset of chronic graft versus host disease, observed in about 30% of cases, lowers the QoL to 0.85: therefore, we set up an average QALY value of 0.9 for post-allogeneic HSCT survival 26. Analyses with and without adjustment for QoL were performed independently. The Markov models were implemented with the *msm* package for R (R Development Core Team 2009) 28 freely available from http://CRAN.R-project.org/package=msm.

## Results

### Outcome of MDS patients classified according to IPSS and WPSS

**Figure 2:**
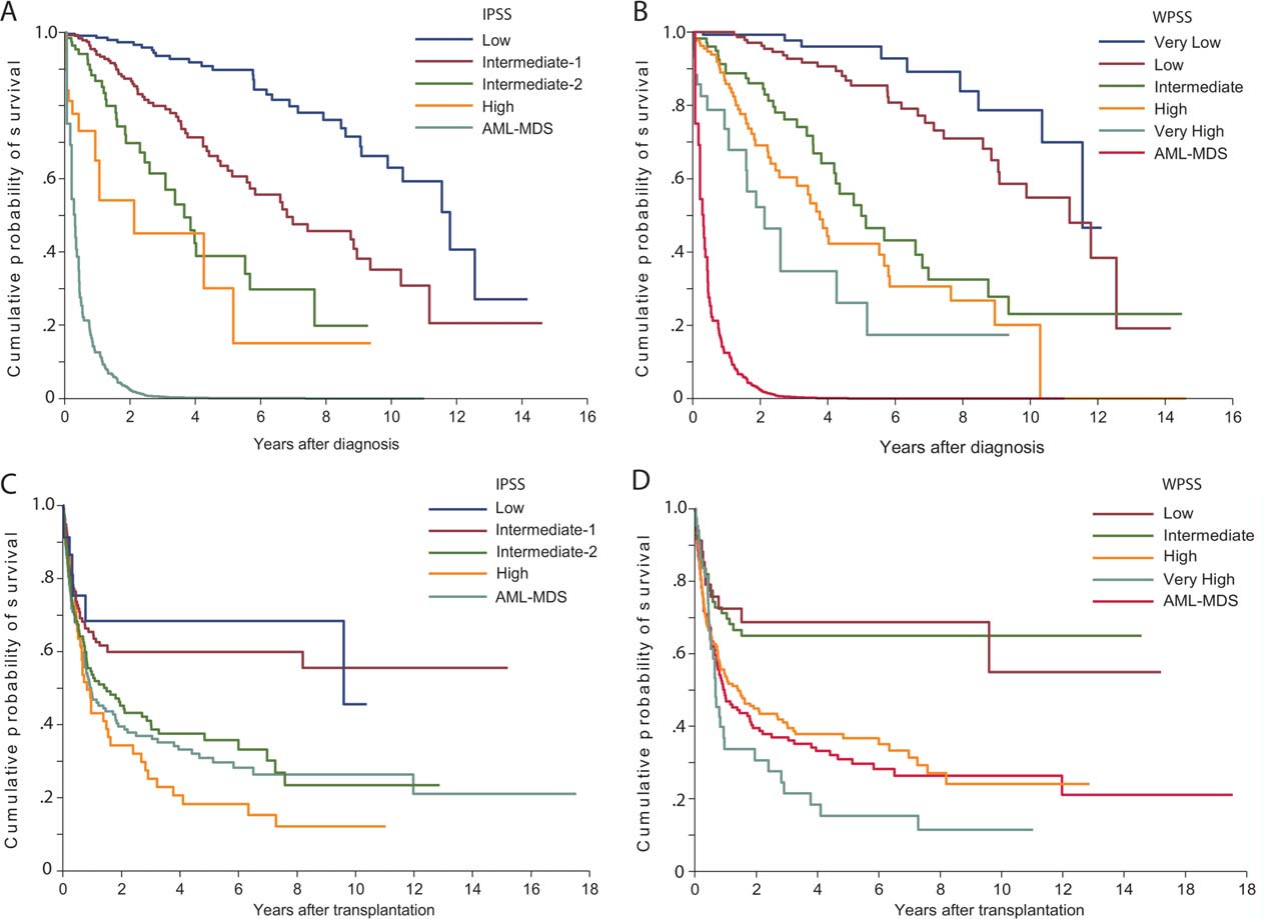
Overall survival of MDS patients. Upper part: overall survival of patients belonging to the Pavia cohort stratified according to time-dependent IPSS (A) or time-dependent WPSS (B). Lower part: overall survival of patients belonging to the GITMO cohort stratified according to IPSS (C) or WPSS (D) scores evaluated at the time of allogeneic HSCT. [Color figure can be viewed in the online issue, which is available at http://wileyonlinelibrary.com.]

In the Pavia cohort, IPSS and WPSS, both analyzed as time-dependent covariates, significantly stratified the probability of survival of MDS patients (*P* < 0.001), as shown in Fig. 2A,B. The cumulative incidences of disease progression, transplantation, and death, analyzed as competing risks for each IPSS and WPSS category, are reported in Supporting Information Fig. 2.

Compared with the Pavia cohort, the GITMO cohort was younger (*P* < 0.001) and included many more subjects with higher IPSS or WPSS risk (*P* < 0.001). IPSS and WPSS at the time of transplantation significantly stratified posttransplantation survival (*P* < 0.001), as shown in Fig. 2C,D.

**Figure 3:**
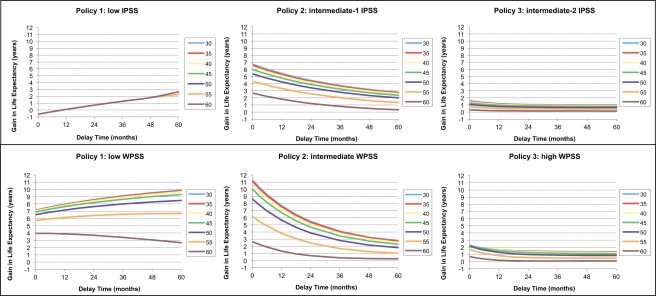
Gain in expected survival since diagnosis according to IPSS and WPSS models under different transplant policies with respect to a nontransplantation policy. We assumed that the MDS patient was classified as low IPSS or very low WPSS risk at the time of diagnosis. Each policy was then evaluated for a set of different ages at diagnosis (between 30 and 65 years, with 5-year intervals, as shown in the boxes) and for different waiting times *t* (between 0 and 60 months since entering any disease state). [Color figure can be viewed in the online issue, which is available at http://wileyonlinelibrary.com.]

In an exploratory multivariate survival analysis, type of donor (HLA-identical sibling versus matched unrelated donor) and conditioning regimen (standard versus reduced-intensity) did not significantly affect posttransplantation survival (*P* = 0.064 and *P* = 0.757, respectively; *P* = 0.759 for interaction). Therefore, all types of donor and conditioning regimen were included in the subsequent decision analysis.

### Decision analysis

We fitted two models of the clinical course of MDS, where patients were stratified according to the IPSS and WPSS, respectively. We first analyzed the goodness of fit of the IPSS- and the WPSS-based Markov models by comparing the estimated survival curves to the Kaplan-Meier estimates. No major lack of fit was detected in either model, as illustrated in Supporting Information Fig. 3.

We then focused on the effect of allogeneic HSCT on survival. In the lower IPSS and WPSS risk groups, the HRs after transplantation were very high due to the risk of NRM, as compared with a relatively low mortality in nontransplanted patients. This was not observed in higher IPSS and WPSS risk categories, in which mortality in nontransplanted subjects was much higher. The HRs associated with transplantation is reported in Supporting Information Table II. The expected survival after diagnosis according to the IPSS and WPSS models was calculated for different transplant policies to assess the optimal transplantation timing.

### IPSS-based transplantation policies

Using the IPSS model, we analyzed the following three policies: (i) policy 1: to perform transplantation in state 1 (low IPSS risk) at *t* months since diagnosis (range 0–60 months) or immediately in case of disease progression before the planned delay time *t*; (ii) policy 2: not to perform transplantation in state 1 and do it in state 2 (intermediate-1 IPSS risk) at *t* months since entering this state (range 0–60 months) or immediately in case of disease progression before the planned delay time *t*; (iii) policy 3: not to perform transplantation in state 1 or 2, and do it in state 3 (intermediate-2 IPSS risk) at *t* months since entering this state (range 0–60 months) or immediately in case of disease progression before the planned delay time *t*. Each policy was evaluated for a set of different ages at diagnosis (between 30 and 65 years with 5-year intervals), and patients lost eligibility to transplantation at 65 years of age.

Gains or losses in life expectancy estimated with respect to a nontransplantation policy are reported in Fig. 3 and Table II, while the obtained life expectancy point estimates under different policies are listed in Supporting Information Table III. Life expectancy increased with increasing delay time of transplantation according to policy 1, but it was overall lower than that estimated according to policy 2 (to delay transplant until progression to intermediate-1 IPSS risk). After progression to this latter risk, life expectancy progressively decreased while delaying transplantation. Delaying transplantation after progression to intermediate-2 IPSS (policy 3) resulted in lower values for life expectancy compared to those estimated according to policy 2, irrespective of the delay time *t*.

**TABLE II:** Estimated Gains or Losses in Life Expectancy (Years) According to Different Transplantation Policies and Variable Patient's Age

IPSS-based transplantation policies		Patient's age (years)			WPSS-based transplantation policies		Patient's age (years)		
			
	Delay time (months)	40	50	60		Delay time (months)	40	50	60
Policy 1: transplantation in low IPSS risk	0	−0.60	−0.60	−0.60	Policy 1: transplantation in low WPSS risk	0	7.05	6.53	3.97
	12	0.09	0.09	0.09		12	7.82	7.16	3.88
	24	0.71	0.71	0.71		24	8.44	7.64	3.68
	48	1.80	1.80	1.80		48	9.34	8.27	3.05
	60	2.27	2.27	2.65		60	9.67	8.48	2.67
Policy 2: transplantation in intermediate-1 IPSS risk	0	6.37	5.38	2.67	Policy 2: transplantation in intermediate WPSS risk	0	10.77	8.66	2.67
	12	5.11	4.25	1.82		12	7.29	5.67	1.33
	24	4.18	3.41	1.21		24	5.15	3.88	0.68
	48	2.95	2.32	0.51		48	3.04	2.18	0.28
	60	2.58	2.00	0.32		60	2.55	1.81	0.25
Policy 3: transplantation in intermediate-2 IPSS risk	0	1.44	1.09	0.32	Policy 3: transplantation in high WPSS risk	0	2.24	2.18	0.73
	12	1.08	0.79	0.19		12	1.63	1.30	0.20
	24	0.96	0.69	0.16		24	1.39	1.00	0.09
	48	0.91	0.65	0.16		48	1.28	0.87	0.10
	60	0.90	0.65	0.15		60	1.26	0.86	0.09

### WPSS-based transplantation policies

Using the WPSS model, we analyzed three policies: (i) policy 1: not to perform transplantation in state 1 (very low WPSS risk) and do it in state 2 (low WPSS risk) at *t* months since entering this state (range 0–60 months) or immediately in case of disease progression before the planned delay time *t*; (ii) policy 2: not to perform transplantation in state 1 or 2, and do it in state 3 (intermediate WPSS risk) at *t* months since entering the state (range 0–60 months) or immediately in case of disease progression before the planned delay time *t*; (iii) policy 3: not to perform transplantation in state 1 to 3, and do it in state 4 (high WPSS) at *t* months since entering the state (range 0–60 months) or immediately in case of disease progression before the planned delay time *t*. Each policy was evaluated for a set of different ages at diagnosis (between 30 and 65 years with 5-year intervals), and patients lost eligibility to transplantation at 65 years of age.

Under policy 1 (transplantation in low WPSS risk), life expectancy increased with increasing delay in transplantation, at least for patients under the age of 60 (Supporting Information Table III): the younger the patient, the higher the gain in life expectancy (Fig. 3). Under policy 2 (transplantation in intermediate WPSS risk), the gain in life expectancy obtained with immediate transplantation in intermediate state (*t* = 0) was comparable to that obtained with a 60-month delay under policy 1. Delaying transplantation in intermediate WPSS risk resulted in loss of life expectancy at any age (Table II and Fig. 3). Delaying transplantation after progression to high WPSS risk (policy 3) resulted in a lower life expectancy compared to that estimated under policy 2 (Table II and Fig. 3).

### QoL adjusted life expectancy

Adjustment for QoL did not affect the outcome of transplantation for any of the IPSS and WPSS risk groups (see QALY-adjusted life expectancies in Supporting Information Table III).

### IPSS-versus WPSS-based transplantation strategies

**Figure 4:**
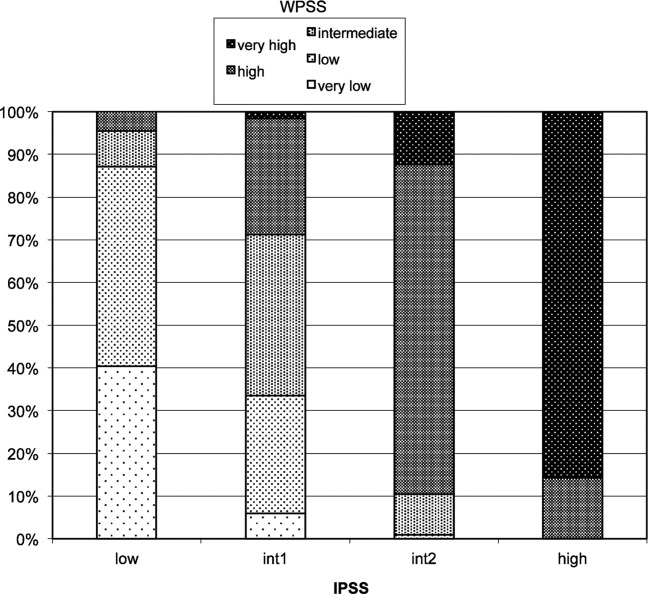
Distribution of WPSS risks within the IPSS risk groups in the Pavia patients that were eligible for transplantation (age <65 years). Within the low IPSS risk group, WPSS identifies a subgroup of patients (13%, first column on the left) with multilineage dysplasia and/or transfusion-dependency that are classified as intermediate or high WPSS risk and would benefit from early transplantation. Within the intermediate-1 IPSS risk group, WPSS identifies about one third (34%, second column from left) of patients with no blast excess and without poor risk cytogenetics that are classified as very low or low WPSS risk and may benefit from delayed transplantation.

To evaluate the extent to which making a decision based on WPSS versus IPSS may lead to a different transplantation strategy in lower MDS risks, we cross-tabulated the distribution of patients in the Pavia cohort according to their IPSS and WPSS scores. Among patients who at any point during follow-up were classified as low IPSS risk and were therefore candidates to a delayed transplantation according to an IPSS-based strategy, 13% had an intermediate or high WPSS score (Fig. 4), and would consequently benefit from an immediate transplantation according to a WPSS-based strategy. This subset specifically included patients with multilineage dysplasia and/or transfusion-dependency.

Among patients who were classified as intermediate-1 IPSS risk and were therefore candidates to immediate transplantation according to an IPSS-based strategy, 34% had a very low or low WPSS risk (Fig. 4), and would consequently be candidates to delayed transplantation according to a WPSS-based strategy. This subgroup included patients without excess of blasts or without poor risk cytogenetics.

We then compared the life expectancy estimated under the best IPSS-based strategy (i.e., transplantation immediately after entering the intermediate-1 risk group) with that estimated under the best WPSS-based strategy (i.e., transplantation immediately after entering the intermediate risk group). Overall, there was a 4-year gain in life expectancy using the WPSS-based policy, and this gain was maximized in younger patients (Table II).

## Discussion

Patients with MDS may be diagnosed at any stage of the disease, and their life expectancy may vary from several years to few months according to the disease-related risk, which can be assessed using the IPSS or WPSS as shown in Fig. 2 10,14. The risk stage is also very relevant to the outcome of allogeneic HSCT, since patients with lower IPSS or WPSS risks have much better posttransplantation survival than those with higher risks, as shown in Fig. 2C,D. These findings confirm previous observations that transplantation early after diagnosis of MDS is associated with the most favorable outcome 16,17,29–31.

The decision analysis by the IBMTR based on a discrete-time Markov model concluded that life expectancy of patients with low or intermediate-1 IPSS risk at diagnosis was higher when transplantation was delayed but performed before the progression of AML 19. This conclusion is difficult to reconcile with the survival data reported in Fig. 2C, showing that MDS patients transplanted at the intermediate-1 IPSS risk have a much better outcome than patients transplanted at the intermediate-2 IPSS risk stage. We believe that the IBMTR analysis had weaknesses mainly related to the unavailability of longitudinal clinical data. In fact, clinical features of the nontransplantation cohort were only available at diagnosis and at the time of leukemic evolution or death. Therefore, the model adopted did not account for disease progression from lower to higher risk stages, which is typical of the natural course of disease and may significantly affect clinical outcome.

This study included a cohort of untreated MDS patients with detailed longitudinal clinical data previously used to develop the WPSS 14. On this basis, we adopted a continuous-time Markov approach to model the natural course of disease, while data on transplanted patients from the GITMO registry were used to estimate the effect of transplantation on survival 16. The output form of the Markov model allowed us to estimate life expectancies under different transplantation policies, and to compare them with a nontransplantation policy.

Using IPSS for risk assessment, the estimated life expectancy increased when transplantation was delayed until progression from low to intermediate-1 risk, and then decreased with further risk increments. These findings are at variance with those of the IBMTR study 19, mainly in that our analysis did no show any advantage in delaying transplantation in the intermediate-1 risk group. These observation has relevant clinical implications, as patients with intermediate-1 IPSS accounted for about half of all MDS patients reported to IBMTR in 2002 31.

We then tested a transplantation policy based on WPPS, which was shown to improve the prognostic stratification of low-grade MDS and significantly stratify the outcome of transplantation 16. Using WPSS, the estimated life expectancy was maximized when transplantation was delayed until progression from the very low or low risk to the intermediate risk, and then decreased. Compared with the IPSS, the use of WPPS provided a better evaluation of the optimal timing of transplantation. In fact, the estimated life expectancy provided by the best WPSS-based strategy was 4 year greater that that provided by the best IPSS-based strategy. Within the low IPSS risk, WPSS identifies a subgroup of patients with multilineage dysplasia and/or transfusion-dependency who may benefit from early transplantation. Conversely, within the intermediate-1 IPSS risk, WPSS identifies patients without excess blasts or with favorable cytogenetics who may benefit from a delayed transplantation strategy.

In this study, started 3 years ago, we did not consider the recently developed IPSS-R, which is based on a novel cytogenetic stratification 12. The IPSS-R needs to be validated in the transplantation setting before an ad hoc Markov model can be developed. It should be noted, however, that it has been recently shown that the 5-group cytogenetic risk classification of the IPSS-R has greater discriminating power for post-transplantation relapse and mortality than the IPSS cytogenetic risk classification 32.

The availability of novel disease-modifying therapies might affect the conclusions of our decision analysis. In a randomized clinical trial, azacitidine was shown to improve survival of patients with higher-risk MDS not eligible for allogeneic HSCT 33. However, more than 70% of patients in this trial were 65 or older, thus showing little overlap with the population included in our analysis. Moreover, a decision analysis has been recently conducted comparing reduced-intensity allogeneic-HSCT versus non-transplantation approaches including azacitidine in MDS patients aged 60–70 years 34. This study showed that early transplantation offers survival benefit for intermediate-2/high IPSS MDS, that is, the condition for which azacitidine has been approved. There is no evidence so far that this drug can significantly improve survival of MDS patients belonging to lower risk groups.

**Figure 5:**
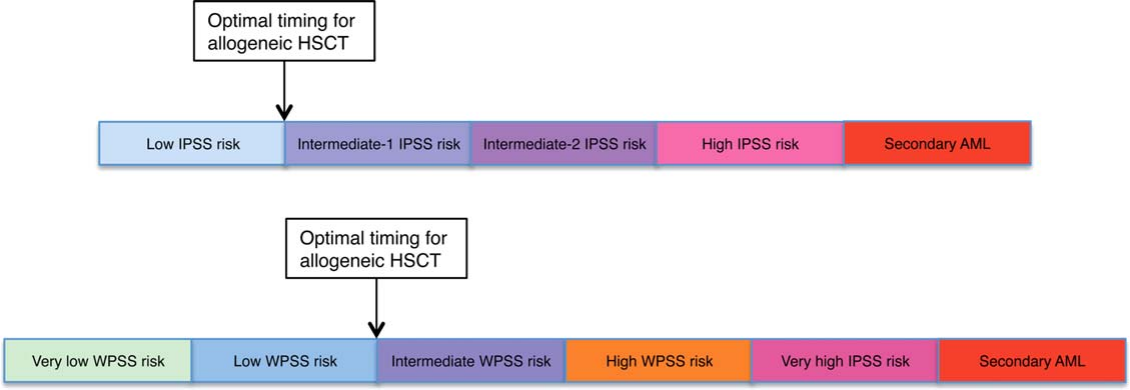
Schematic representation of the natural history of myelodysplastic syndrome according to IPSS or WPSS risk stratification. The Markov decision analysis performed in this study indicates that allogeneic hematopoietic stem cell transplantation offers optimal survival benefit when it is performed early in intermediate-1 IPSS risk or intermediate WPSS risk stage, respectively. [Color figure can be viewed in the online issue, which is available at http://wileyonlinelibrary.com.]

In summary, the findings of our study, based on modeling of the natural course of MDS, indicate that a delayed transplantation strategy is advisable for patients with early disease, that is, those with low IPSS and very low or low WPSS risk. Allogeneic HSCT should instead be offered to eligible patients belonging to intermediate risk categories, in particular to those with intermediate-1 IPSS or intermediate WPSS risk, since this strategy offers the best survival benefit (Fig. 5). Finally, modeling decision analysis on WPSS versus IPSS allows a better estimation of the optimal timing of transplantation, especially because the WPSS accounts for transfusion dependency, an independent negative prognostic factor for transplantation outcome.

## Appendix

The following institutions in Italy contributed to the trial: Division of Hematology, Ospedale “S.S. Antonio e Biagio” Alessandria (A. Levis); Division of Hematology, Ospedali Riuniti, Bergamo (A. Rambaldi); Institute of Hematology and Clinical Oncology L.A. Seragnoli, Ospedale S. Orsola-Malpighi, University of Bologna, Bologna (G. Bandini); Department of Hematology, Ospedale Regionale, Bolzano (M. Casini); Division of Hematology, Spedali Civili, Brescia (G. Rossi); Division of Hematology and Bone Marrow Transplant Center, Ospedale Oncologico A. Businco, Cagliari (E. Angelucci, D. Baronciani); Bone Marrow Transplantation Unit, Ospedale R. Binagli, University of Cagliari, Cagliari (G. La Nasa); Division of Hematology and Bone Marrow Transplantation, Ospedale Ferrarotto” Catania (G. Milone); Division of Hematology, Ospedale S. Croce e Carlo, Cuneo (N. Mordini); Department of Hematology, Ospedale Careggi, University of Florence, Firenze (S. Guidi, A. Bosi); Division of Hematology, Ospedale S. Martino, Genova (A. Bacigalupo, MT. Van Lint), Hematology-Bone Marrow Transplantation Unit, Istituto Nazionale dei Tumori, University of Milan, Milan (P. Corradini, R. Milani), Division of Hematology Ospedale Cà Granda Niguarda, Milan (E. Morra, P. Marenco); Department of Hematology, Fondazione IRCCS Ospedale Maggiore Policlinico, Mangiagalli e Regina Elena, Milan (G. Lambertenghi Deliliers, F. Onida); Hematology and BMT Unit, Department of Oncology, San Raffaele Scientific Institute, Milan (F. Ciceri, M. Bernardi); Transplantation Unit, Department of Oncology-Hematology, IRCCS Clinica Humanitas, Rozzano (L. Castagna); Department of Oncology and Hematology, University of Modena and Reggio Emilia, Modena (F. Narni); Division of Hematology and Transplant Unit, Ospedale S. Gerardo, University of Milano-Bicocca, Monza (P. Pioltelli), Division of Hematology, University of Napoli Federico II Medical School, Napoli (C. Selleri); Division of Hematology and Transplant Unit, Ospedale V. Cervello, Palermo (R. Scimè); Division of Hematology, University of Palermo, Palermo (E. Iannitto); Department of Oncology, Hematology Unit, Ospedale La Maddalena” Palermo (M. Musso), Depaertment of Hematology Oncology, Fondazione IRCCS Policlinico San Matteo, University of Pavia, Pavia (E.P. Alessandrino); Pediatric Hematology-Oncology, University of Pavia, Fondazione IRCCS Policlinico “S. Matteo”, Pavia (F. Locatelli, M. Zecca); Department of Hematology, University of Perugia, Policlinico “Monteluce”, Perugia (F. Martelli); Hematology and Transplant Centre, Ospedale S. Salvatore, Pesaro (Visani G); Department of Hematology, Ospedale Civile, Pescara (P. Di Bartolomeo); Oncology and Hematology Department, Ospedale Guglielmo da Saliceto, Piacenza (L. Cavanna); Division of Hematology, Univeristy of Pisa, Pisa (F. Papineschi); Transplant Unit A. Neri, Ospedale Bianchi-Melacrino-Morelli, Reggio Calabria (G. Messina); Hematology Unit, Arcispedale S. Maria Nuova, Reggio Emilia (F. Merli); Division of Hematology, Department of Cellular Biotechnologies and Hematology, University La Sapienza (A.P. Iori, R. Foà); Hematology and Stem Cell Transplantation Unit Ospedale S. Camillo, Roma (A. Locasciulli, I. Majolino); Hematology, University S. Cuore, Roma (P. Chiusolo, G. Leone); Rome Transplant Network, Department of Hematology, Stem Cell Transplant Unit, Policlinico Tor Vergata (W. Arcese, R. Cerretti); Unit of Hematology and Bone Marrow Transplantation, IRCCS Casa Sollievo della Sofferenza, S. Giovanni Rotondo (A.M. Carella, N. Cascavilla); Institute of Hematology, Ospedale Nord, Taranto (P. Mazza); Division of Hematology, Ospedale S. Giovanni Battista, Torino (M. Falda); Division of Hematology, University of Torino, Ospedale S. Giovanni Battista, Torino (B. Bruno, M. Boccadoro); Division of Hematology and Bone Marrow Transplantation, University of Udine, Udine (R. Fanin, M. Cerno); Department of Hematology, Ospedale S. Bortolo, Vicenza (R. Raimondi).

## References

[1] Cazzola M, Malcovati L (2005). Myelodysplastic syndromes-coping with ineffective hematopoiesis. N Engl J Med.

[2] Malcovati L, Della Porta MG, Pascutto C (2005). Prognostic factors and life expectancy in myelodysplastic syndromes classified according to WHO criteria: a basis for clinical decision making. J Clin Oncol.

[3] Bejar R, Stevenson K, Abdel-Wahab O (2011). Clinical effect of point mutations in myelodysplastic syndromes. N Engl J Med.

[4] Yoshida K, Sanada M, Shiraishi Y (2011). Frequent pathway mutations of splicing machinery in myelodysplasia. Nature.

[5] Papaemmanuil E, Cazzola M, Boultwood J (2011). Somatic SF3B1 mutation in myelodysplasia with ring sideroblasts. N Engl J Med.

[6] Malcovati L, Papaemmanuil E, Bowen DT (2011). Clinical significance of SF3B1 mutations in myelodysplastic syndromes and myelodysplastic/myeloproliferative neoplasms. Blood.

[7] Graubert TA, Shen D, Ding L (2012). Recurrent mutations in the U2AF1 splicing factor in myelodysplastic syndromes. Nat Genet.

[8] Bejar R, Stevenson KE, Caughey BA (2012). Validation of a prognostic model and the impact of mutations in patients with lower-risk myelodysplastic syndromes. J Clin Oncol.

[9] Thol F, Kade S, Schlarmann C (2012). Frequency and prognostic impact of mutations in SRSF2, U2AF1, and ZRSR2 in patients with myelodysplastic syndromes. Blood.

[10] Greenberg P, Cox C, LeBeau MM (1997). International scoring system for evaluating prognosis in myelodysplastic syndromes. Blood.

[11] Appelbaum FR (2011). The role of hematopoietic cell transplantation as therapy for myelodysplasia. Best Pract Res Clin Haematol.

[12] Greenberg PL, Tuechler H, Schanz J (2012). Revised international prognostic scoring system for myelodysplastic syndromes. Blood.

[13] Vardiman JW, Harris NL, Brunning RD (2002). The World Health Organization (WHO) classification of the myeloid neoplasms. Blood.

[14] Malcovati L, Germing U, Kuendgen A (2007). Time-dependent prognostic scoring system for predicting survival and leukemic evolution in myelodysplastic syndromes. J Clin Oncol.

[15] Malcovati L, Della Porta MG, Strupp C (2011). Impact of the degree of anemia on the outcome of patients with myelodysplastic syndrome and its integration into the WHO classification-based Prognostic Scoring System (WPSS). Haematologica.

[16] Alessandrino EP, Della Porta MG, Bacigalupo A (2008). WHO classification and WPSS predict posttransplantation outcome in patients with myelodysplastic syndrome: A study from the Gruppo Italiano Trapianto di Midollo Osseo (GITMO). Blood.

[17] Lim Z, Brand R, Martino R (2010). Allogeneic hematopoietic stem-cell transplantation for patients 50 years or older with myelodysplastic syndromes or secondary acute myeloid leukemia. J Clin Oncol.

[18] Kroger N (2012). Allogeneic stem cell transplantation for elderly patients with myelodysplastic syndrome. Blood.

[19] Cutler CS, Lee SJ, Greenberg P (2004). A decision analysis of allogeneic bone marrow transplantation for the myelodysplastic syndromes: Delayed transplantation for low-risk myelodysplasia is associated with improved outcome. Blood.

[20] Swerdlow SH, Campo E, Harris NL (2008). WHO classification of tumours of haematopoietic and lymphoid tissues.

[21] Bennett JM, Catovsky D, Daniel MT (1982). Proposals for the classification of the myelodysplastic syndromes. Br J Haematol.

[22] Alessandrino EP, Della Porta MG, Bacigalupo A (2010). Prognostic impact of pre-transplantation transfusion history and secondary iron overload in patients with myelodysplastic syndrome undergoing allogeneic stem cell transplantation: a GITMO study. Haematologica.

[23] Kalbfleisch JD, Lawless JF, Vollmer WM (1983). Estimation in Markov models from aggregate data. Biometrics.

[24] Kay R (1986). A Markov model for analysing cancer markers and disease states in survival studies. Biometrics.

[25] Sung L, Buckstein R, Doyle JJ (2003). Treatment options for patients with acute myeloid leukemia with a matched sibling donor: A decision analysis. Cancer.

[26] Chiodi S, Spinelli S, Ravera G (2000). Quality of life in 244 recipients of allogeneic bone marrow transplantation. Br J Haematol.

[27] Bush NE, Donaldson GW, Haberman MH (2000). Conditional and unconditional estimation of multidimensional quality of life after hematopoietic stem cell transplantation: A longitudinal follow-up of 415 patients. Biol Blood Marrow Transplant.

[28] Jackson CH (2011). Multi-state models for panel data: The msm package for R. Journal of Statistical Software.

[29] Anderson JE, Appelbaum FR, Schoch G (1996). Allogeneic marrow transplantation for refractory anemia: A comparison of two preparative regimens and analysis of prognostic factors. Blood.

[30] de Witte T, Hermans J, Vossen J (2000). Haematopoietic stem cell transplantation for patients with myelo-dysplastic syndromes and secondary acute myeloid leukaemias: A report on behalf of the Chronic Leukaemia Working Party of the European Group for Blood and Marrow Transplantation (EBMT). Br J Haematol.

[31] Sierra J, Perez WS, Rozman C (2002). Bone marrow transplantation from HLA-identical siblings as treatment for myelodysplasia. Blood.

[32] Deeg HJ, Scott BL, Fang M (2012). Five-group cytogenetic risk classification, monosomal karyotype, and outcome after hematopoietic cell transplantation for MDS or acute leukemia evolving from MDS. Blood.

[33] Fenaux P, Mufti GJ, Hellstrom-Lindberg E (2009). Efficacy of azacitidine compared with that of conventional care regimens in the treatment of higher-risk myelodysplastic syndromes: a randomised, open-label, phase III study. Lancet Oncol.

[34] Koreth J, Pidala J, Perez WS The role of reduced-intensity conditioning allogeneic hematopoietic stem cell transplantation in older patients with de-novo myelodysplastic syndromes: An international collaborative decision analysis. J Clin Oncol.

