# Supplementary material for: Optimal timing of allogeneic hematopoietic stem cell transplantation in patients with myelodysplastic syndrome
Source: Am J Hematol. 2013 Apr 20;88(7):581–8. doi: 10.1002/ajh.23458 (PMC3736162; doi:10.1002/ajh.23458)
Supplement: Supplementary file 1 [file ajh0088-0581-SD1.pdf]

Alessandrino et al. Optimal timing of allogeneic hematopoietic stem cell transplantation in patients with myelodysplastic syndrome. Am J Hematol 2013. DOI: 10.1002/ajh.23458

Supporting Information

Supporting Information Methods

Patients and study design

Supporting Information Table I. Transplant-related features of patients belonging to the GITMO cohort.

Transplant related features	WHO classification	
	MDS	AML evolving from MDS
Time from diagnosis to allogeneic HSCT (months; months, range)	10 (1-343)	8 (1-410)
Type of donor:*		N=131 (94%)
Sibling	213 (63%)	89 (64%)
Matched unrelated donor (MUD)	124 (37%)	51 (36%)
Source of hematopoietic stem cells		
Peripheral blood / cord blood	220 (65%)	77 (55%)
Bone marrow	117 (35%)	63 (45%)
Remission-induction chemotherapy		
Patients untreated	239/337 (71%)	15/140 (11%)
Patients treated	98/337 (29%)	125/140 (89%)
Patients achieving complete remission	51/98 (52%)	54/125 (43%)
Conditioning regimen**		
Standard conditioning	211 (63%)	91 (65%)
Reduced-intensity conditioning (RIC)	126 (37%)	49 (35%)

* Criteria for selection of HLA-matched unrelated donors before 2002 included low-resolution typing for HLA class I (A, B) and high-resolution typing for HLA-DRB1, whereas since 2002 criteria included high-resolution typing for both HLA class I (A-C) and class II alleles (DRB1/3/4/5, DQA1, DPB1).
** Most frequent conditioning regimens included: total body irradiation (TBI) and cyclophosphamide (20% of cases), TBI and fludarabine (8%), busulphan and cyclophosphamide (30%), thiotepa and cyclophosphamide (24%), and thiotepa and fludarabine (11%). For most patients, graft-versus-host disease (GVHD) prophylaxis was combined cyclosporine and methotrexate.

Descriptive statistics and survival analysis

Numerical variables are summarized by median and range, categorical variables by count and relative frequency (%) in each category. Survival analyses were performed with the Kaplan-Meier product limit method. For patients in the Pavia cohort, overall survival (OS) was defined as the time (months) between the date of diagnosis and the date of death (for cases) or last follow up (for censored patients). Patients who underwent allogeneic transplantation, acute myeloid leukemia (AML)-like chemotherapy or treatment with hypomethylating agents were censored at the time of the therapeutic procedure. IPSS and WPSS risk were analyzed as time-dependent variables. Comparisons between Kaplan-Meier curves were made using the Gehan Wilcoxon test. A competing risk analysis (Kalbfleisch-Prentice) was performed to estimate the cumulative incidence of disease progression to a higher risk category, of receiving allogeneic HSCT and of death for all causes in each IPSS and WPSS category.

In the GITMO cohort, OS was defined as the time between transplantation and death (from any cause) or last follow up (for censored observations). When estimating non-relapse mortality (NRM), any death in the absence of disease relapse was considered an event. The probability of relapse was estimated considering treatment as a failure at the time of hematologic relapse according to standardized criteria. The cumulative incidence of relapse and NRM was estimated with a competing risks approach (Kalbfleisch-Prentice).

Analyses were performed using Stata 11.2 SE software (StataCorp LP, College Station, TX, USA).

Markov model

A multi-state model describes how an individual moves between a series of states in time. Markov models are multi-state models based on Markov processes, i.e. a stochastic process (a mathematical model for a random development in time) with the property that the probability of moving to a particular state in the future only depends on the present state, and not on past states. In other words, in Markov models the past influences the future only via the present. This may seem too strong an assumption, but in many instances it is at least plausible and, above all, it allows a relatively simple implementation of multi-state models.

Markov processes are frequently used to model chronic diseases, because model states have a natural interpretation in terms of staged progression. A commonly-used model represents a series of successively more severe disease stages or states, and an "absorbing" state, often death. The patient enters the model at a given time 0 (e.g., the time of diagnosis) and then may advance into or recover from adjacent disease stages, or die at any disease stage. The "life" of a patient is the time spent jumping between states, i.e. the time elapsed between time 0 and the patient's final transition to the absorbing state. By iterating the model a large number of times, it is possible to estimate the life expectancy of a patient.

In discrete-time Markov processes, transition from one state to the next is only allowed at discrete time-points, and a probability of transition is estimated for each allowed transition and for each time point. Discrete-time Markov processes are commonly used as decision models because they can be implemented rather easily. However, the discretization of time (e.g. allowing "jumps" between states only at fixed time points such as every given number of days, months or years) may be an oversimplification of the underlying process.

Continuous-time multi-state models allow transitions between states to take place at any point in time, and not just at the start of a discrete cycle (such as a month or year). The next state to which the individual moves, and the time of the change, are governed by a set of transition intensities, which may also depend on the time of the process, or more generally on a set of individual-specific or time-varying explanatory variables. In more detail, observations of the state $S_n(t)$ are made on a number of individuals n at arbitrary times t, which may vary between individuals. The stages of disease may be modeled as a homogeneous continuous-time Markov process governed by a matrix Q of transition intensities q_{ij} representing the instantaneous risk of progression from the ith to the jth state. Under this model, the time spent in state i has an exponential distribution with mean $-1/q_{ii}$ and the probability that the next state is j is $-q_{ij}/q_{ii}$. Multi-state Markov models may be fitted to data with irregular observation times using freely available software (Jackson CH. Multi-state models for panel data: The msm package for R. *J Stat Soft*. 2011;38(8):1-28).

In the case of MDS, each risk category (either IPSS or WPSS) of the disease may be represented by a state in the model, with death as absorbing state (i.e., a state in which transitions to other states are not allowed). A transition intensity is then estimated for each possible transition from a state to a higher disease risk or to death. In this setting, allogeneic

HSCT may be modeled as a time-dependent categorical covariate. At any point in time it may assume 3 possible values: no transplantation performed so far; transplantation preformed less than three months before (to allow for excess of mortality due to transplant-related causes); transplantation performed more that three months before. The effect of HSCT on survival is then estimated as a hazard ratio with respect to the “non transplantation” category.

The multi-state Markov models used for MDS are illustrated in Supporting Information Figure 1. IPSS (A) and WPSS (B) risk scores were adopted as time-dependent indicators of the natural course of the disease. The expected survival (expected time spent by a subject in the model before reaching the absorbing state) under different transplant policies was calculated algebraically for the fitted Markov models. These calculations were validated by microsimulation, and a confidence interval was obtained by bootstrap resampling (Supporting Information Table III).

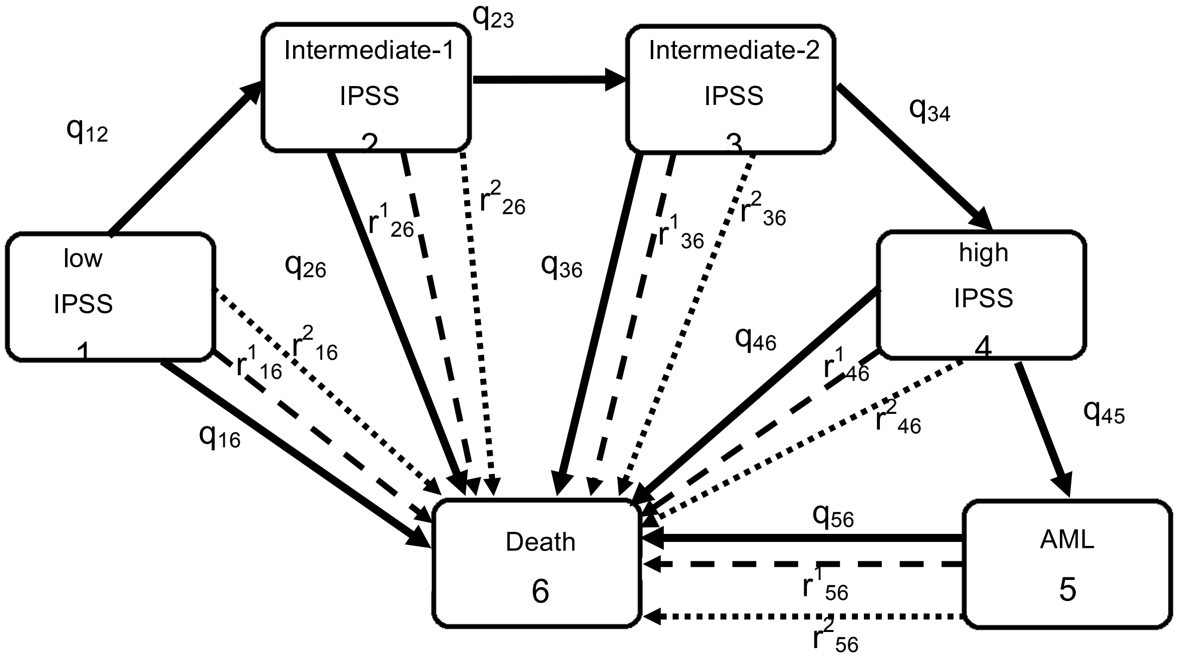

$q_{i,i+1}$ = transition intensity from i^{th} state to the next; i=1,…,5 (disease progression)

q_{i6} = mortality rate in state i when not transplanted

r^1_{i6} = hazard ratio of death in state i up to 3 months after allogeneic HSCT vs. q_{i6}

r^2_{i6} = hazard ratio of death in state i from 3 months after allogeneic HSCT onwards vs. q_{i6}

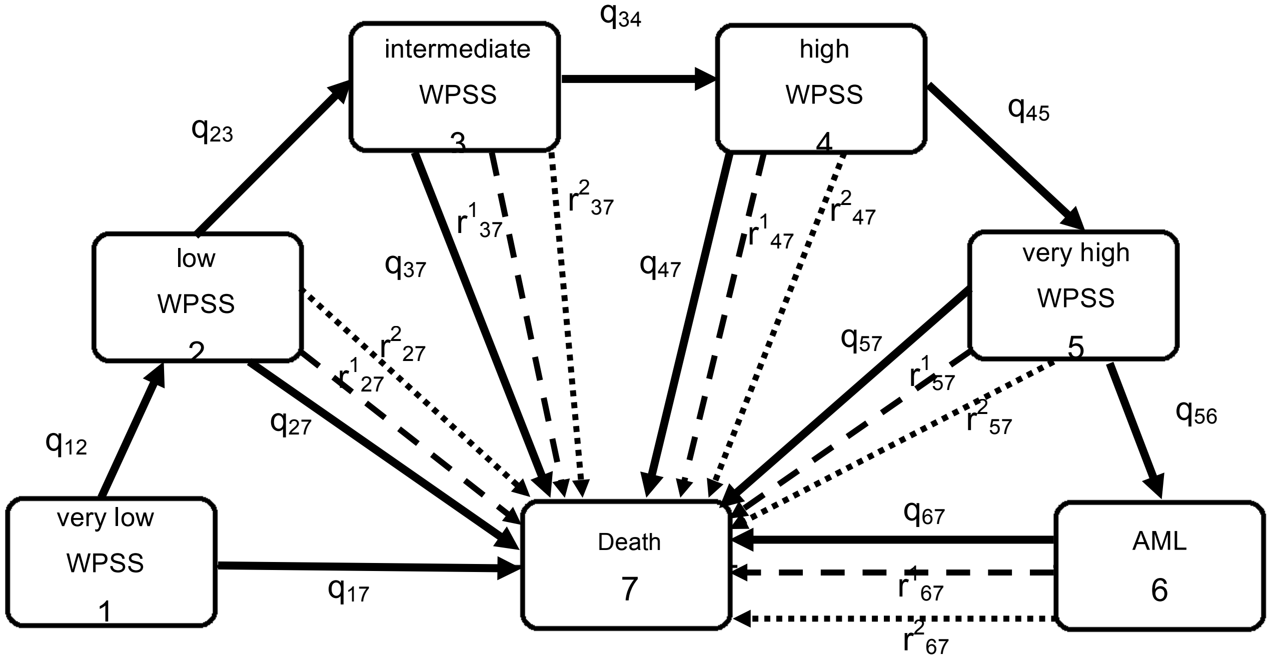

$q_{i\,i+1}$ = transition intensity from i^{th} state to the next; i=1,…,6 (disease progression)

q_{i7} = mortality rate in state i when not transplanted

r^1_{i7} = hazard ratio of death in state i up to 3 months after allogeneic HSCT vs. q_{i7}

r^2_{i7} = hazard ratio of death in state i from 3 months after allogeneic HSCT onwards vs. q_{i7}

Supporting Information Figure 1. Markov continuous-time multi-state models of the natural history of MDS based on IPSS (upper part) and WPSS (lower part).

Supporting Information Results

(A)

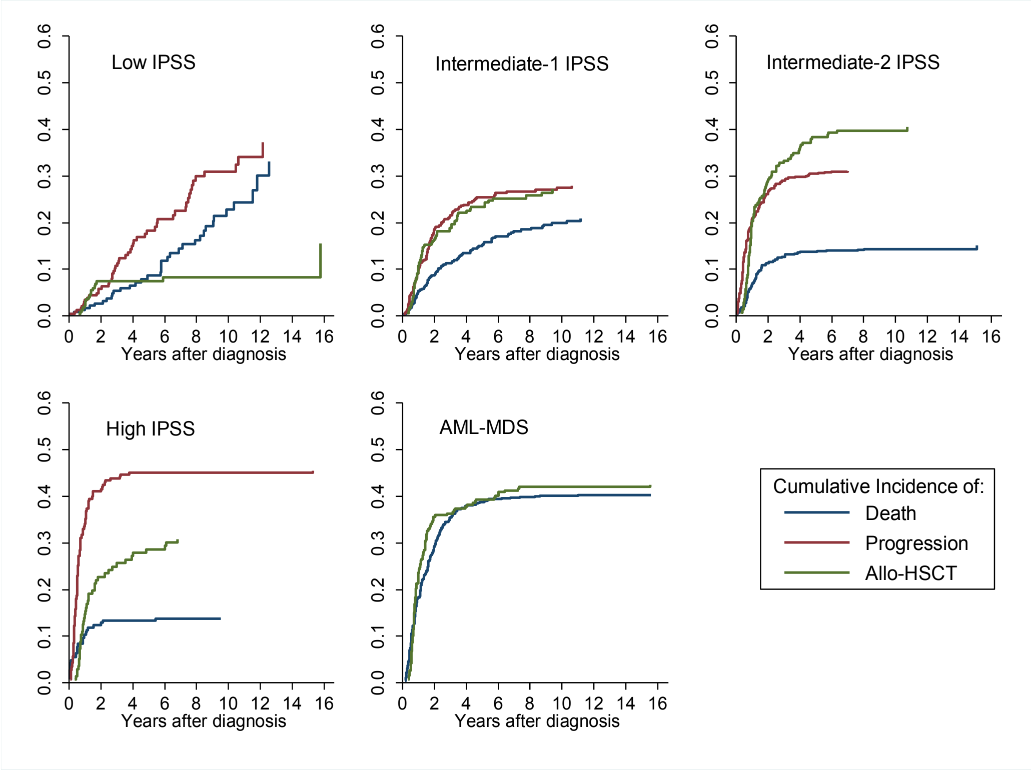

(B)

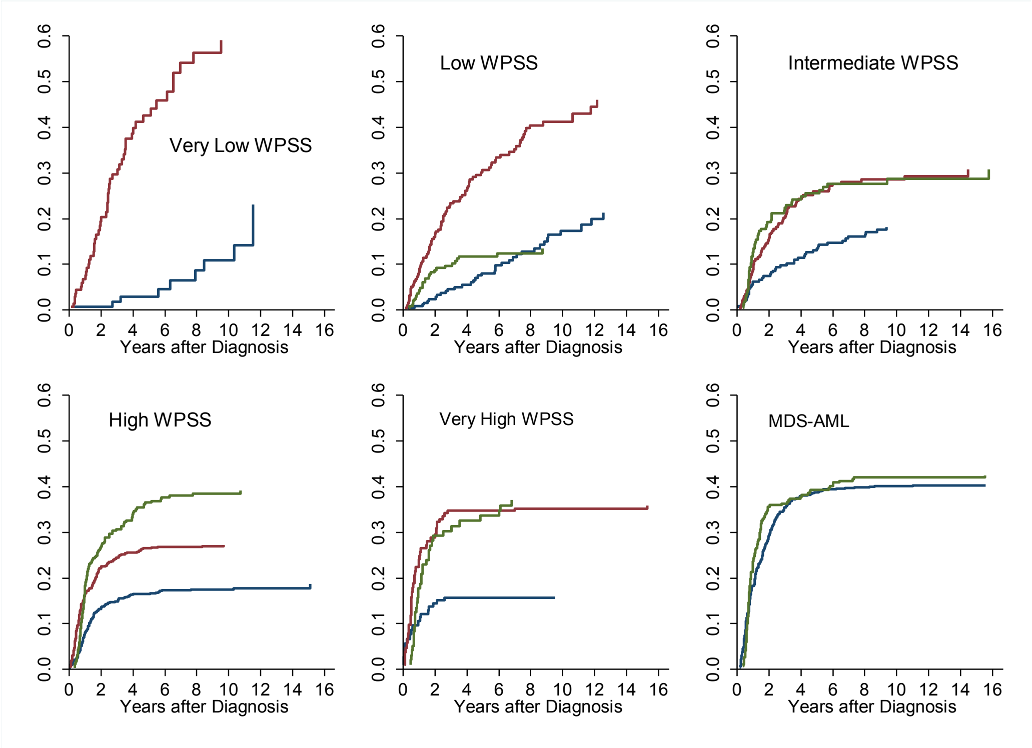

Supporting Information Figure 2. Competing risk analysis of the cumulative incidence of disease progression, death and treatment with allogeneic HSCT in each IPSS (A) and WPSS (B) risk group in the Pavia cohort.

Supporting Information Table II. Summary of fitted continuous-time Markov models for IPSS (A) and WPSS (B). For each state, the maximum likelihood and 95% CI of the expected length of stay (conditional upon surviving to that state) are reported, along with the transition intensity to a higher risk score and to death. As to transition intensities, their absolute value is of little practical use, being an instantaneous risk of transition. The comparison of transition intensities between different states, however, may be interpreted as a relative risk of transition. For example, the transition intensity to death (instantaneous risk of death) in the high IPSS risk category is 7-fold the intensity in the low IPSS risk.

(A) IPSS model: Expected survival in each state and transition intensities.

	Expected years in state (given survival to state)			Transition intensity to next state			Transition intensity to death		
IPSS risk	Point estimate	95% CI		Point estimate	95% CI		Point estimate	95% CI	
Low	10.5	8.5	13.0	q_{12}=0.0069	0.0053	0.0088	q_{16}=0.0010	0.0005	0.0023
Intermediate-1	3.5	3.0	4.0	q_{23}=0.0223	0.0191	0.0260	q_{26}=0.0017	0.0008	0.0037
Intermediate-2	0.9	0.8	1.1	q_{34}=0.0885	0.0759	0.1031	q_{36}=0.0015	0.0003	0.0085
High	0.1	0.1	0.2	q_{45}=0.5556	0.4406	0.7005	q_{46}=0.0068	0.0005	0.0934
AML	1.2	1.0	1.4				q_{56}=0.0680	0.0577	0.0800

(A) IPSS model: Hazard ratio for death in each state, according to time elapsed since transplantation.

	Up to 3 months			After 3 months		
IPSS risk	Point estimate	95% CI		Point estimate	95% CI	
Low	r^1_{16}=53.51	13.43	213.18	r^2_{16}= 4.62	1.15	18.52
Intermediate-1	r^1_{26}=40.99	16.75	100.26	r^2_{26}= 2.98	1.23	7.23
Intermediate-2	r^1_{36}=51.91	8.83	305.16	r^2_{36}=10.63	1.84	61.39
High	r^1_{46}=11.22	0.76	165.73	r^2_{46}= 3.98	0.28	56.33
Post-MDS AML	r^1_{56}= 1.30	0.89	1.88	r^2_{56}= 0.25	0.19	0.34

(B) WPSS model: Expected survival in each state and transition intensities.

	Expected years in state (given survival to state)			Transition intensity to next state			Transition intensity to death		
WPSS risk	Point estimate	95% CI		Point estimate	95% CI		Point estimate	95% CI	
Very low	6.2	4.8	8.0	q_{12}=0.0131	0.0101	0.0170	q_{17}=0.0003	0.0000	0.0024
Low	4.8	4.0	5.7	q_{23}=0.0169	0.0142	0.0202	q_{27}=0.0005	0.0001	0.0025
Intermediate	2.1	1.7	2.5	q_{34}=0.0381	0.0318	0.0458	q_{37}=0.0020	0.0006	0.0063
High	1.1	1.0	1.3	q_{45}=0.0705	0.0602	0.0826	q_{47}=0.0037	0.0016	0.0090
Very high	0.2	0.2	0.3	q_{56}=0.3475	0.2795	0.4321	q_{57}=0.0042	0.0002	0.0819
AML	1.1	0.9	1.3				q_{67}=0.0781	0.0664	0.0918

(B) WPSS model: Hazard ratio for death in each state, according to time elapsed since transplantation.

	Up to 3 months			After 3 months		
WPSS risk	Point estimate	95% CI		Point estimate	95% CI	
Low	r^1_{27}=95.48	13.77	662.07	r^2_{27}=9.72	1.55	60.78
Intermediate	r^1_{37}=28.92	8.01	104.39	r^2_{37}=2.14	0.60	7.63
High	r^1_{47}=24.30	9.64	61.30	r^2_{47}=3.92	1.58	9.71
Very high	r^1_{57}=12.08	0.55	264.05	r^2_{57}=6.41	0.32	129.28
AML	r^1_{67}= 1.13	0.77	1.64	r^2_{67}=0.22	0.17	0.30

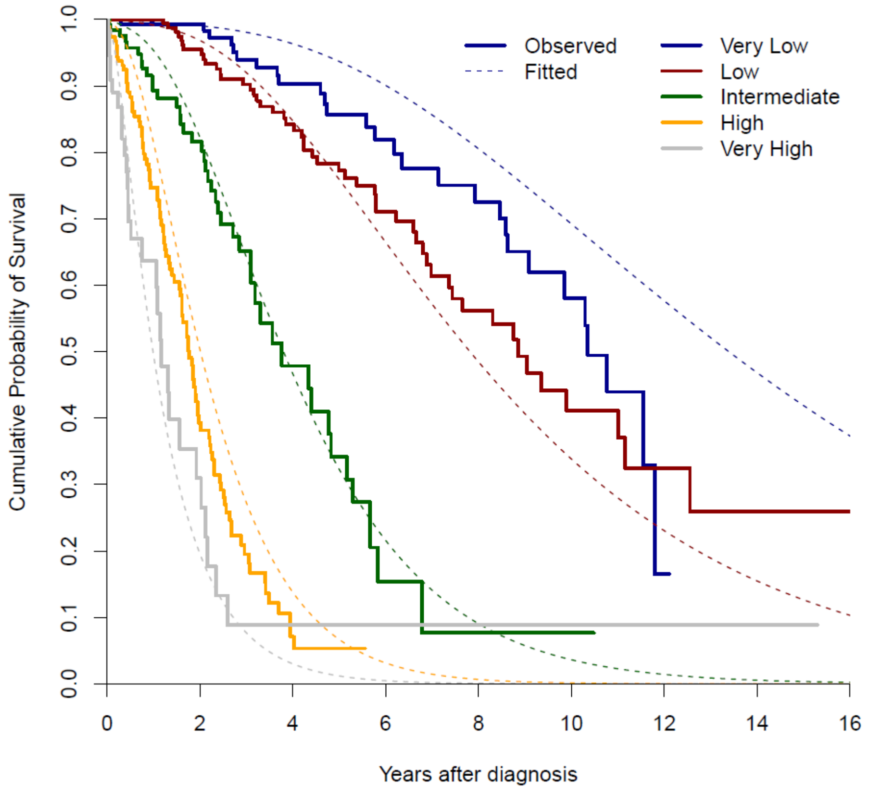

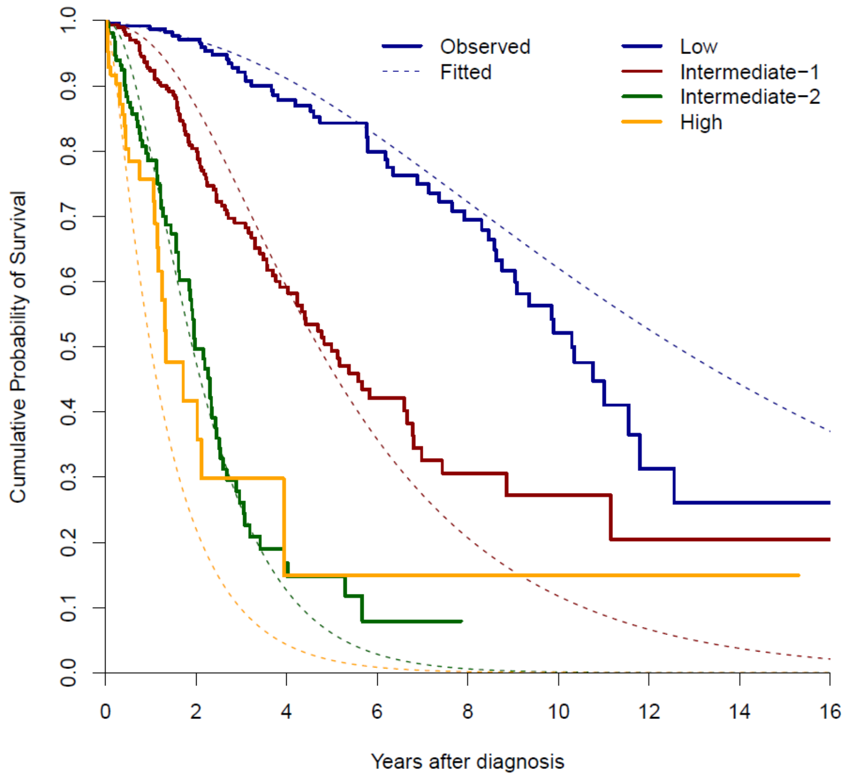

Supporting Information Figure 3. Goodness of fit of Markov model (dotted lines) for natural course of MDS according to IPSS (upper part) and WPSS (lower part) relative to observed survival (Kaplan-Meier analysis, solid lines) in patients receiving supportive care.

Supporting Information Table III. Expected survival (95% CI) in years according to transplantation policy, age at diagnosis and delay time *t* of transplant (months). (A) IPSS model; (B) WPSS model. Quality of life adjusted estimates for both models are shown in italics.

(A)

		Age at diagnosis							
Policy	Delay time t	30	35	40	45	50	55	60	65
Low IPSS risk	0	14.77 (4.71,44.42)<br>*13.29 (4.24,39.98)*	14.77 (4.71,44.42)<br>*13.29 (4.24,39.98)*	14.77 (4.71,44.42)<br>*13.29 (4.24,39.98)*	14.77 (4.71,44.42)<br>*13.29 (4.24,39.98)*	14.77 (4.71,44.42)<br>*13.29 (4.24,39.98)*	14.77 (4.71,44.42)<br>*13.29 (4.24,39.98)*	14.77 (4.71,44.42)<br>*13.29 (4.24,39.98)*	15.37 (13.04,17.66)<br>*14.46 (12.26,16.64)*
	1	14.83 (4.84,44.24)<br>*13.35 (4.36,39.82)*	14.83 (4.84,44.24)<br>*13.35 (4.36,39.82)*	14.83 (4.84,44.24)<br>*13.35 (4.36,39.82)*	14.83 (4.84,44.24)<br>*13.35 (4.36,39.82)*	14.83 (4.84,44.24)<br>*13.35 (4.36,39.82)*	14.83 (4.84,44.24)<br>*13.35 (4.36,39.82)*	14.83 (4.84,44.24)<br>*13.35 (4.36,39.82)*	15.37 (13.04,17.66)<br>*14.46 (12.26,16.64)*
	3	14.95 (5.1,43.96)<br>*13.46 (4.6,39.58)*	14.95 (5.1,43.96)<br>*13.46 (4.6,39.58)*	14.95 (5.1,43.96)<br>*13.46 (4.6,39.58)*	14.95 (5.1,43.96)<br>*13.46 (4.6,39.58)*	14.95 (5.1,43.96)<br>*13.46 (4.6,39.58)*	14.95 (5.1,43.96)<br>*13.46 (4.6,39.58)*	14.95 (5.1,43.96)<br>*13.46 (4.6,39.58)*	15.37 (13.04,17.66)<br>*14.46 (12.26,16.64)*
	6	15.12 (5.5,43.36)<br>*13.63 (4.98,39.04)*	15.12 (5.5,43.36)<br>*13.63 (4.98,39.04)*	15.12 (5.5,43.36)<br>*13.63 (4.98,39.04)*	15.12 (5.5,43.36)<br>*13.63 (4.98,39.04)*	15.12 (5.5,43.36)<br>*13.63 (4.98,39.04)*	15.12 (5.5,43.36)<br>*13.63 (4.98,39.04)*	15.12 (5.5,43.36)<br>*13.63 (4.98,39.04)*	15.37 (13.04,17.66)<br>*14.46 (12.26,16.64)*
	12	15.46 (6.24,42.49)<br>*13.96 (5.66,38.29)*	15.46 (6.24,42.49)<br>*13.96 (5.66,38.29)*	15.46 (6.24,42.49)<br>*13.96 (5.66,38.29)*	15.46 (6.24,42.49)<br>*13.96 (5.66,38.29)*	15.46 (6.24,42.49)<br>*13.96 (5.66,38.29)*	15.46 (6.24,42.49)<br>*13.96 (5.66,38.29)*	15.46 (6.24,42.49)<br>*13.96 (5.66,38.29)*	15.37 (13.04,17.66)<br>*14.46 (12.26,16.64)*
	18	15.78 (6.95,41.52)<br>*14.27 (6.32,37.44)*	15.78 (6.95,41.52)<br>*14.27 (6.32,37.44)*	15.78 (6.95,41.52)<br>*14.27 (6.32,37.44)*	15.78 (6.95,41.52)<br>*14.27 (6.32,37.44)*	15.78 (6.95,41.52)<br>*14.27 (6.32,37.44)*	15.78 (6.95,41.52)<br>*14.27 (6.32,37.44)*	15.78 (6.95,41.52)<br>*14.27 (6.32,37.44)*	15.37 (13.04,17.66)<br>*14.46 (12.26,16.64)*
	24	16.08 (7.64,40.8)<br>*14.57 (6.97,36.82)*	16.08 (7.64,40.8)<br>*14.57 (6.97,36.82)*	16.08 (7.64,40.8)<br>*14.57 (6.97,36.82)*	16.08 (7.64,40.8)<br>*14.57 (6.97,36.82)*	16.08 (7.64,40.8)<br>*14.57 (6.97,36.82)*	16.08 (7.64,40.8)<br>*14.57 (6.97,36.82)*	16.08 (7.64,40.8)<br>*14.57 (6.97,36.82)*	15.37 (13.04,17.66)<br>*14.46 (12.26,16.64)*
	36	16.65 (8.85,38.95)<br>*15.12 (8.1,35.19)*	16.65 (8.85,38.95)<br>*15.12 (8.1,35.19)*	16.65 (8.85,38.95)<br>*15.12 (8.1,35.19)*	16.65 (8.85,38.95)<br>*15.12 (8.1,35.19)*	16.65 (8.85,38.95)<br>*15.12 (8.1,35.19)*	16.65 (8.85,38.95)<br>*15.12 (8.1,35.19)*	16.65 (8.85,38.95)<br>*15.12 (8.1,35.19)*	15.37 (13.04,17.66)<br>*14.46 (12.26,16.64)*
	48	17.17 (9.98,37.48)<br>*15.62 (9.15,33.9)*	17.17 (9.98,37.48)<br>*15.62 (9.15,33.9)*	17.17 (9.98,37.48)<br>*15.62 (9.15,33.9)*	17.17 (9.98,37.48)<br>*15.62 (9.15,33.9)*	17.17 (9.98,37.48)<br>*15.62 (9.15,33.9)*	17.17 (9.98,37.48)<br>*15.62 (9.15,33.9)*	17.17 (9.98,37.48)<br>*15.62 (9.15,33.9)*	15.37 (13.04,17.66)<br>*14.46 (12.26,16.64)*
	60	17.64 (11.01,36.04)<br>*16.08 (10.11,32.64)*	17.64 (11.01,36.04)<br>*16.08 (10.11,32.64)*	17.64 (11.01,36.04)<br>*16.08 (10.11,32.64)*	17.64 (11.01,36.04)<br>*16.08 (10.11,32.64)*	17.64 (11.01,36.04)<br>*16.08 (10.11,32.64)*	17.64 (11.01,36.04)<br>*16.08 (10.11,32.64)*	18.02 (15.2,20.75)<br>*16.81 (14.19,19.34)*	15.37 (13.04,17.66)<br>*14.46 (12.26,16.64)*
Int-1 IPSS risk	0	22.07 (17.21,28.11)<br>*20.39 (15.93,25.85)*	21.92 (17.16,27.8)<br>*20.25 (15.91,25.59)*	21.7 (17.06,27.27)<br>*20.06 (15.82,25.17)*	21.32 (16.91,26.53)<br>*19.73 (15.72,24.49)*	20.71 (16.61,25.39)<br>*19.19 (15.43,23.47)*	19.67 (16.12,23.55)<br>*18.27 (14.99,21.8)*	18 (15.2,20.75)<br>*16.79 (14.19,19.35)*	15.33 (13.03,17.67)<br>*14.42 (12.25,16.64)*
	1	21.95 (17.16,27.86)<br>*20.28 (15.93,25.6)*	21.79 (17.11,27.58)<br>*20.15 (15.91,25.37)*	21.58 (17.01,27.06)<br>*19.96 (15.81,24.97)*	21.2 (16.87,26.33)<br>*19.63 (15.67,24.3)*	20.61 (16.55,25.19)<br>*19.1 (15.36,23.26)*	19.57 (16.08,23.34)<br>*18.19 (14.95,21.63)*	17.92 (15.16,20.62)<br>*16.72 (14.15,19.24)*	15.33 (13.03,17.67)<br>*14.42 (12.25,16.64)*
	3	21.71 (17.09,27.33)<br>*20.07 (15.87,25.2)*	21.55 (17.03,27.06)<br>*19.93 (15.81,24.91)*	21.35 (16.92,26.62)<br>*19.76 (15.72,24.5)*	20.98 (16.77,25.89)<br>*19.43 (15.55,23.87)*	20.4 (16.44,24.77)<br>*18.92 (15.3,22.87)*	19.38 (16,22.98)<br>*18.02 (14.89,21.29)*	17.76 (15.08,20.37)<br>*16.58 (14.07,19.02)*	15.33 (13.03,17.67)<br>*14.42 (12.25,16.64)*
	6	21.37 (16.92,26.68)<br>*19.78 (15.71,24.57)*	21.22 (16.86,26.39)<br>*19.64 (15.67,24.34)*	21.02 (16.77,25.95)<br>*19.47 (15.57,23.94)*	20.67 (16.61,25.24)<br>*19.16 (15.43,23.32)*	20.1 (16.34,24.15)<br>*18.66 (15.2,22.33)*	19.12 (15.88,22.5)<br>*17.78 (14.78,20.88)*	17.54 (14.97,20.04)<br>*16.39 (13.97,18.7)*	15.33 (13.03,17.67)<br>*14.42 (12.25,16.64)*
	12	20.77 (16.72,25.43)<br>*19.26 (15.53,23.48)*	20.63 (16.67,25.18)<br>*19.13 (15.49,23.26)*	20.44 (16.58,24.78)<br>*18.97 (15.4,22.92)*	20.11 (16.41,24.14)<br>*18.67 (15.27,22.35)*	19.58 (16.14,23.14)<br>*18.21 (15.02,21.46)*	18.64 (15.64,21.63)<br>*17.37 (14.59,20.12)*	17.15 (14.72,19.53)<br>*16.05 (13.77,18.28)*	15.33 (13.03,17.67)<br>*14.42 (12.26,16.64)*
	18	20.25 (16.5,24.4)<br>*18.8 (15.37,22.57)*	20.11 (16.45,24.16)<br>*18.68 (15.33,22.36)*	19.94 (16.37,23.8)<br>*18.53 (15.24,22.03)*	19.63 (16.22,23.2)<br>*18.26 (15.1,21.53)*	19.13 (15.94,22.29)<br>*17.81 (14.86,20.75)*	18.24 (15.45,20.92)<br>*17.02 (14.43,19.55)*	16.82 (14.47,19.15)<br>*15.75 (13.54,17.95)*	15.33 (13.03,17.67)<br>*14.42 (12.26,16.64)*
	24	19.79 (16.33,23.55)<br>*18.41 (15.21,21.85)*	19.67 (16.29,23.32)<br>*18.3 (15.17,21.66)*	19.51 (16.18,23.01)<br>*18.15 (15.09,21.36)*	19.22 (16.02,22.47)<br>*17.89 (14.94,20.86)*	18.74 (15.76,21.61)<br>*17.47 (14.7,20.14)*	17.9 (15.28,20.41)<br>*16.73 (14.28,19.07)*	16.54 (14.27,18.79)<br>*15.51 (13.35,17.65)*	15.33 (13.03,17.67)<br>*14.43 (12.26,16.64)*

	36	19.07 (15.99,22.24)<br>*17.77 (14.91,20.71)*	18.96 (15.93,22.06)<br>*17.68 (14.86,20.55)*	18.81 (15.85,21.78)<br>*17.54 (14.79,20.28)*	18.56 (15.69,21.33)<br>*17.32 (14.66,19.89)*	18.12 (15.4,20.66)<br>*16.93 (14.41,19.29)*	17.36 (14.91,19.7)<br>*16.25 (13.96,18.44)*	16.14 (13.87,18.39)<br>*15.15 (13.01,17.3)*	15.33 (13.03,17.67)<br>*14.43 (12.26,16.64)*
	48	18.52 (15.64,21.42)<br>*17.3 (14.63,20)*	18.43 (15.59,21.24)<br>*17.21 (14.57,19.84)*	18.29 (15.5,20.99)<br>*17.09 (14.51,19.6)*	18.06 (15.36,20.61)<br>*16.89 (14.37,19.26)*	17.66 (15.1,20.04)<br>*16.53 (14.13,18.77)*	16.96 (14.6,19.21)<br>*15.9 (13.66,18.03)*	15.85 (13.57,18.09)<br>*14.9 (12.74,17.03)*	15.34 (13.03,17.67)<br>*14.43 (12.26,16.64)*
	60	18.11 (15.39,20.84)<br>*16.94 (14.4,19.51)*	18.03 (15.35,20.69)<br>*16.87 (14.37,19.37)*	17.91 (15.26,20.47)<br>*16.76 (14.3,19.16)*	17.69 (15.11,20.13)<br>*16.57 (14.17,18.86)*	17.33 (14.86,19.63)<br>*16.24 (13.9,18.41)*	16.67 (14.35,18.92)<br>*15.65 (13.45,17.77)*	15.65 (13.37,17.92)<br>*14.72 (12.54,16.88)*	15.33 (13.03,17.67)<br>*14.43 (12.26,16.64)*
Int-2 IPSS risk	0	16.96 (14.34,19.43)<br>*16.06 (13.59,18.41)*	16.92 (14.3,19.34)<br>*16 (13.57,18.32)*	16.81 (14.26,19.22)<br>*15.89 (13.53,18.2)*	16.68 (14.17,19.04)<br>*15.75 (13.44,18.01)*	16.46 (14.01,18.76)<br>*15.54 (13.29,17.76)*	16.12 (13.76,18.38)<br>*15.24 (13.06,17.42)*	15.69 (13.35,17.93)<br>*14.84 (12.68,17.03)*	15.37 (13.02,17.65)<br>*14.54 (12.38,16.78)*
	1	16.91 (14.32,19.35)<br>*16.01 (13.58,18.33)*	16.87 (14.29,19.27)<br>*15.95 (13.56,18.25)*	16.76 (14.25,19.14)<br>*15.84 (13.5,18.13)*	16.63 (14.15,18.98)<br>*15.71 (13.43,17.95)*	16.41 (13.99,18.69)<br>*15.5 (13.29,17.72)*	16.09 (13.73,18.32)<br>*15.22 (13.04,17.4)*	15.67 (13.33,17.92)<br>*14.82 (12.67,17.01)*	15.37 (13.02,17.65)<br>*14.54 (12.38,16.78)*
	3	16.82 (14.27,19.22)<br>*15.93 (13.55,18.2)*	16.78 (14.23,19.14)<br>*15.88 (13.52,18.12)*	16.68 (14.18,19.01)<br>*15.77 (13.47,18.01)*	16.55 (14.1,18.85)<br>*15.65 (13.39,17.86)*	16.35 (13.95,18.59)<br>*15.44 (13.23,17.64)*	16.04 (13.71,18.27)<br>*15.17 (13.01,17.35)*	15.64 (13.31,17.9)<br>*14.79 (12.64,16.99)*	15.37 (13.02,17.65)<br>*14.54 (12.38,16.78)*
	6	16.71 (14.19,19.07)<br>*15.84 (13.46,18.07)*	16.67 (14.16,19)<br>*15.78 (13.44,18)*	16.58 (14.12,18.88)<br>*15.68 (13.4,17.91)*	16.46 (14.02,18.73)<br>*15.56 (13.31,17.79)*	16.27 (13.88,18.5)<br>*15.37 (13.19,17.58)*	15.98 (13.66,18.19)<br>*15.11 (12.97,17.3)*	15.6 (13.29,17.87)<br>*14.76 (12.62,16.96)*	15.37 (13.03,17.65)<br>*14.54 (12.38,16.78)*
	12	16.56 (14.1,18.92)<br>*15.71 (13.4,17.94)*	16.53 (14.08,18.86)<br>*15.66 (13.37,17.87)*	16.45 (14.03,18.75)<br>*15.57 (13.33,17.79)*	16.34 (13.96,18.59)<br>*15.46 (13.24,17.66)*	16.16 (13.82,18.39)<br>*15.28 (13.11,17.47)*	15.89 (13.6,18.13)<br>*15.04 (12.9,17.2)*	15.56 (13.25,17.82)<br>*14.72 (12.59,16.92)*	15.37 (13.03,17.65)<br>*14.54 (12.38,16.78)*
	18	16.47 (14.03,18.85)<br>*15.64 (13.33,17.88)*	16.44 (14.01,18.78)<br>*15.58 (13.31,17.82)*	16.37 (13.96,18.68)<br>*15.5 (13.28,17.74)*	16.27 (13.88,18.55)<br>*15.4 (13.19,17.61)*	16.09 (13.74,18.36)<br>*15.23 (13.06,17.42)*	15.85 (13.56,18.09)<br>*15 (12.88,17.18)*	15.55 (13.23,17.81)<br>*14.7 (12.57,16.9)*	15.37 (13.02,17.65)<br>*14.55 (12.38,16.79)*
	24	16.43 (13.95,18.81)<br>*15.59 (13.28,17.86)*	16.4 (13.93,18.74)<br>*15.54 (13.27,17.8)*	16.33 (13.89,18.65)<br>*15.46 (13.22,17.71)*	16.23 (13.83,18.51)<br>*15.36 (13.14,17.57)*	16.06 (13.71,18.33)<br>*15.2 (13.03,17.4)*	15.83 (13.51,18.08)<br>*14.98 (12.86,17.17)*	15.53 (13.22,17.8)<br>*14.69 (12.57,16.9)*	15.37 (13.02,17.65)<br>*14.55 (12.38,16.79)*
	36	16.38 (13.9,18.79)<br>*15.55 (13.21,17.84)*	16.35 (13.89,18.73)<br>*15.5 (13.19,17.78)*	16.29 (13.85,18.63)<br>*15.42 (13.17,17.69)*	16.19 (13.79,18.49)<br>*15.33 (13.11,17.56)*	16.03 (13.67,18.32)<br>*15.18 (13.01,17.38)*	15.81 (13.48,18.06)<br>*14.96 (12.84,17.17)*	15.53 (13.21,17.8)<br>*14.69 (12.56,16.9)*	15.37 (13.03,17.65)<br>*14.55 (12.38,16.79)*
	48	16.37 (13.89,18.76)<br>*15.54 (13.2,17.83)*	16.33 (13.87,18.72)<br>*15.49 (13.18,17.77)*	16.28 (13.84,18.62)<br>*15.41 (13.15,17.68)*	16.18 (13.77,18.48)<br>*15.32 (13.08,17.56)*	16.02 (13.66,18.31)<br>*15.17 (13,17.38)*	15.8 (13.48,18.05)<br>*14.95 (12.83,17.17)*	15.53 (13.21,17.8)<br>*14.68 (12.56,16.9)*	15.37 (13.03,17.65)<br>*14.55 (12.38,16.79)*
	60	16.36 (13.88,18.76)<br>*15.53 (13.19,17.83)*	16.33 (13.87,18.72)<br>*15.48 (13.17,17.76)*	16.27 (13.82,18.61)<br>*15.41 (13.14,17.69)*	16.18 (13.76,18.48)<br>*15.32 (13.08,17.56)*	16.02 (13.66,18.3)<br>*15.16 (13,17.37)*	15.8 (13.47,18.05)<br>*14.95 (12.83,17.17)*	15.52 (13.21,17.8)<br>*14.68 (12.56,16.9)*	15.37 (13.03,17.65)<br>*14.55 (12.38,16.79)*

(B)		Age at diagnosis							
Policy	Delay time t	30	35	40	45	50	55	60	65
Low WPSS risk	0	22.15 (13.22,39.94)<br>*20.55 (12.42,36.54)*	22.12 (13.22,39.81)<br>*20.52 (12.43,36.46)*	22.05 (13.22,39.55)<br>*20.46 (12.44,36.22)*	21.89 (13.22,38.92)<br>*20.33 (12.46,35.7)*	21.53 (13.26,37.65)<br>*20.02 (12.49,34.57)*	20.73 (13.31,34.84)<br>*19.34 (12.55,32.03)*	18.97 (13.45,28.76)<br>*17.81 (12.75,26.67)*	15 (12.52,16.65)<br>*14.4 (12.02,16.04)*
	1	22.23 (13.54,39.75)<br>*20.62 (12.81,36.39)*	22.19 (13.47,39.62)<br>*20.59 (12.69,36.27)*	22.12 (13.43,39.31)<br>*20.53 (12.66,36.04)*	21.96 (13.43,38.71)<br>*20.4 (12.67,35.49)*	21.59 (13.46,37.42)<br>*20.08 (12.69,34.29)*	20.77 (13.5,34.49)<br>*19.38 (12.73,31.76)*	18.97 (13.56,28.49)<br>*17.81 (12.88,26.4)*	15 (12.52,16.65)<br>*14.4 (12.02,16.04)*
	3	22.37 (13.87,39.13)<br>*20.76 (13.09,35.85)*	22.33 (13.79,39)<br>*20.72 (13,35.75)*	22.26 (13.76,38.79)<br>*20.66 (12.99,35.53)*	22.1 (13.77,38.17)<br>*20.52 (13,35.06)*	21.71 (13.79,36.93)<br>*20.19 (13.01,33.9)*	20.85 (13.84,34.11)<br>*19.46 (13.06,31.38)*	18.97 (13.83,27.97)<br>*17.81 (13.11,25.96)*	15 (12.52,16.65)<br>*14.4 (12.02,16.04)*
	6	22.58 (14.41,38.57)<br>*20.96 (13.57,35.42)*	22.53 (14.4,38.44)<br>*20.92 (13.54,35.3)*	22.46 (14.34,38.22)<br>*20.85 (13.51,35.05)*	22.29 (14.35,37.61)<br>*20.7 (13.53,34.52)*	21.87 (14.33,36.28)<br>*20.35 (13.51,33.36)*	20.96 (14.33,33.43)<br>*19.57 (13.52,30.85)*	18.95 (14.13,27.17)<br>*17.81 (13.37,25.26)*	15 (12.52,16.65)<br>*14.4 (12.02,16.04)*
	12	22.95 (15.31,37.6)<br>*21.31 (14.39,34.45)*	22.91 (15.29,37.49)<br>*21.28 (14.37,34.37)*	22.82 (15.27,37.18)<br>*21.19 (14.36,34.16)*	22.63 (15.26,36.57)<br>*21.03 (14.33,33.62)*	22.16 (15.2,35.28)<br>*20.63 (14.29,32.49)*	21.14 (15.07,32.26)<br>*19.75 (14.18,29.79)*	18.88 (14.65,25.81)<br>*17.77 (13.86,24.06)*	15 (12.52,16.65)<br>*14.4 (12.02,16.04)*
	18	23.29 (16.01,36.58)<br>*21.64 (15.04,33.6)*	23.24 (15.97,36.5)<br>*21.6 (15.01,33.49)*	23.15 (15.95,36.22)<br>*21.51 (15,33.29)*	22.93 (15.9,35.5)<br>*21.32 (14.97,32.74)*	22.42 (15.82,34.12)<br>*20.88 (14.88,31.49)*	21.29 (15.63,31.14)<br>*19.91 (14.71,28.81)*	18.79 (14.91,24.47)<br>*17.71 (14.12,22.88)*	15 (12.52,16.65)<br>*14.4 (12.02,16.04)*
	24	23.6 (16.57,35.76)<br>*21.94 (15.54,32.86)*	23.55 (16.55,35.52)<br>*21.89 (15.52,32.77)*	23.44 (16.53,35.25)<br>*21.79 (15.51,32.42)*	23.2 (16.47,34.52)<br>*21.58 (15.47,31.87)*	22.64 (16.35,33.15)<br>*21.09 (15.35,30.58)*	21.42 (16,30.15)<br>*20.04 (15.09,27.88)*	18.68 (15.15,23.33)<br>*17.63 (14.34,21.86)*	15 (12.52,16.65)<br>*14.4 (12.02,16.04)*
	36	24.12 (17.36,34.5)<br>*22.44 (16.32,31.77)*	24.07 (17.35,34.37)<br>*22.38 (16.29,31.63)*	23.94 (17.31,34.02)<br>*22.27 (16.26,31.39)*	23.66 (17.26,33.36)<br>*22.02 (16.19,30.75)*	23 (17.09,31.88)<br>*21.46 (16.06,29.51)*	21.59 (16.64,28.76)<br>*20.22 (15.68,26.7)*	18.39 (15.27,21.79)<br>*17.4 (14.49,20.47)*	15 (12.52,16.65)<br>*14.39 (12.02,16.04)*
	48	24.55 (17.88,34)<br>*22.84 (16.77,31.37)*	24.48 (17.87,33.8)<br>*22.78 (16.76,31.24)*	24.34 (17.83,33.52)<br>*22.65 (16.71,30.91)*	24.02 (17.73,32.76)<br>*22.36 (16.62,30.29)*	23.27 (17.51,31.31)<br>*21.72 (16.47,28.92)*	21.67 (16.88,28.03)<br>*20.32 (15.94,26.03)*	18.05 (15.06,20.89)<br>*17.12 (14.34,19.72)*	15 (12.52,16.65)<br>*14.39 (12.02,16.04)*
	60	24.89 (18.29,34.08)<br>*23.17 (17.16,31.45)*	24.82 (18.28,33.86)<br>*23.1 (17.14,31.31)*	24.67 (18.21,33.51)<br>*22.95 (17.08,30.96)*	24.31 (18.09,32.72)<br>*22.63 (17,30.29)*	23.48 (17.8,31.17)<br>*21.93 (16.75,28.88)*	21.7 (17.07,27.72)<br>*20.35 (16.1,25.81)*	17.67 (14.71,20.41)<br>*16.79 (14.03,19.3)*	15 (12.52,16.65)<br>*14.39 (12.02,16.04)*
Int WPSS risk	0	26.33 (18.6,37.45)<br>*24.56 (17.47,34.61)*	26.15 (18.55,37.06)<br>*24.39 (17.43,34.23)*	25.78 (18.44,36.34)<br>*24.06 (17.33,33.57)*	25.05 (18.15,34.9)<br>*23.43 (17.1,32.27)*	23.67 (17.6,32.17)<br>*22.19 (16.65,29.81)*	21.23 (16.6,27.36)<br>*20 (15.69,25.52)*	17.68 (14.72,20.39)<br>*16.8 (14.04,19.31)*	15.01 (12.52,16.65)<br>*14.43 (12.04,16.06)*
	1	25.95 (18.45,36.58)<br>*24.23 (17.39,33.86)*	25.78 (18.38,36.25)<br>*24.07 (17.36,33.52)*	25.42 (18.26,35.52)<br>*23.75 (17.24,32.87)*	24.7 (18.01,34.17)<br>*23.13 (16.99,31.62)*	23.36 (17.5,31.44)<br>*21.91 (16.54,29.2)*	20.98 (16.47,26.81)<br>*19.78 (15.61,25.02)*	17.53 (14.64,20.11)<br>*16.67 (13.97,19.05)*	15.01 (12.52,16.65)<br>*14.43 (12.04,16.06)*
	3	25.25 (18.21,35.05)<br>*23.61 (17.19,32.5)*	25.09 (18.17,34.73)<br>*23.45 (17.15,32.16)*	24.74 (18.05,34.05)<br>*23.15 (17.05,31.55)*	24.06 (17.8,32.72)<br>*22.55 (16.85,30.32)*	22.78 (17.31,30.18)<br>*21.39 (16.39,28.07)*	20.51 (16.31,25.83)<br>*19.36 (15.45,24.14)*	17.27 (14.48,19.56)<br>*16.42 (13.81,18.63)*	15.01 (12.52,16.65)<br>*14.43 (12.04,16.06)*
	6	24.3 (17.94,32.97)<br>*22.77 (16.96,30.6)*	24.15 (17.89,32.7)<br>*22.62 (16.9,30.31)*	23.82 (17.78,32.12)<br>*22.34 (16.81,29.79)*	23.18 (17.55,30.86)<br>*21.77 (16.6,28.69)*	21.99 (17.03,28.52)<br>*20.69 (16.13,26.59)*	19.88 (16.06,24.48)<br>*18.79 (15.22,22.94)*	16.9 (14.25,18.99)<br>*16.11 (13.6,18.09)*	15.01 (12.52,16.65)<br>*14.43 (12.04,16.07)*
	12	22.72 (17.37,29.62)<br>*21.36 (16.45,27.59)*	22.59 (17.33,29.36)<br>*21.23 (16.39,27.33)*	22.3 (17.23,28.8)<br>*20.98 (16.29,26.86)*	21.73 (17,27.74)<br>*20.48 (16.08,25.88)*	20.68 (16.49,25.75)<br>*19.52 (15.63,24.09)*	18.84 (15.48,22.31)<br>*17.88 (14.75,21.03)*	16.34 (13.84,18.08)<br>*15.62 (13.22,17.32)*	15.01 (12.52,16.65)<br>*14.43 (12.05,16.07)*
	18	21.48 (16.83,27.01)<br>*20.26 (15.93,25.22)*	21.36 (16.78,26.77)<br>*20.14 (15.89,25.02)*	21.1 (16.67,26.31)<br>*19.91 (15.81,24.62)*	20.59 (16.45,25.35)<br>*19.47 (15.58,23.79)*	19.66 (15.99,23.61)<br>*18.62 (15.17,22.21)*	18.06 (15.08,20.74)<br>*17.18 (14.36,19.66)*	15.95 (13.48,17.58)<br>*15.28 (12.93,16.89)*	15.01 (12.52,16.65)<br>*14.43 (12.05,16.07)*
	24	20.5 (16.37,24.94)<br>*19.39 (15.54,23.45)*	20.39 (16.33,24.7)<br>*19.29 (15.49,23.24)*	20.16 (16.2,24.31)<br>*19.08 (15.41,22.83)*	19.7 (16,23.47)<br>*18.69 (15.22,22.09)*	18.89 (15.57,22.02)<br>*17.93 (14.85,20.79)*	17.47 (14.67,19.7)<br>*16.65 (14.03,18.73)*	15.69 (13.25,17.27)<br>*15.04 (12.71,16.61)*	15.01 (12.52,16.65)<br>*14.44 (12.05,16.07)*
	36	19.13 (15.63,22.3)<br>*18.17 (14.9,21.11)*	19.04 (15.6,22.13)<br>*18.09 (14.85,20.95)*	18.85 (15.5,21.79)<br>*17.92 (14.77,20.63)*	18.48 (15.33,21.16)<br>*17.59 (14.6,20.06)*	17.82 (14.94,20.09)<br>*16.99 (14.24,19.1)*	16.69 (14.17,18.46)<br>*15.97 (13.55,17.67)*	15.39 (12.94,16.99)<br>*14.77 (12.42,16.36)*	15.01 (12.52,16.66)<br>*14.44 (12.05,16.07)*

	48	18.3 (15.1,20.77)<br>*17.42 (14.42,19.77)*	18.22 (15.06,20.62)<br>*17.35 (14.39,19.63)*	18.05 (14.98,20.35)<br>*17.21 (14.3,19.39)*	17.74 (14.8,19.85)<br>*16.94 (14.16,18.94)*	17.19 (14.47,19.04)<br>*16.42 (13.85,18.21)*	16.27 (13.79,17.9)<br>*15.59 (13.22,17.21)*	15.29 (12.84,16.88)<br>*14.68 (12.33,16.28)*	15.01 (12.52,16.65)<br>*14.44 (12.06,16.07)*
	60	17.78 (14.77,19.97)<br>*16.96 (14.13,19.05)*	17.71 (14.74,19.86)<br>*16.9 (14.1,18.95)*	17.56 (14.66,19.62)<br>*16.78 (14.03,18.74)*	17.3 (14.5,19.2)<br>*16.54 (13.88,18.37)*	16.82 (14.18,18.55)<br>*16.09 (13.57,17.79)*	16.04 (13.62,17.65)<br>*15.39 (13.04,16.98)*	15.26 (12.8,16.86)<br>*14.65 (12.3,16.26)*	15.01 (12.52,16.65)<br>*14.44 (12.05,16.07)*
High WPSS risk	0	17.09 (14.19,19.12)<br>*16.34 (13.6,18.33)*	17.17 (14.26,19.17)<br>*16.41 (13.65,18.41)*	17.23 (14.35,19.19)<br>*16.5 (13.74,18.43)*	17.29 (14.45,19.14)<br>*16.55 (13.83,18.38)*	17.17 (14.47,18.88)<br>*16.48 (13.88,18.16)*	16.71 (14.16,18.3)<br>*16.06 (13.58,17.61)*	15.72 (13.23,17.31)<br>*15.1 (12.71,16.68)*	14.99 (12.54,16.65)<br>*14.4 (12.04,16.05)*
	1	17.03 (14.19,19.04)<br>*16.29 (13.57,18.24)*	17.1 (14.24,19.07)<br>*16.35 (13.62,18.3)*	17.16 (14.32,19.09)<br>*16.43 (13.68,18.31)*	17.19 (14.37,19.02)<br>*16.46 (13.77,18.26)*	17.05 (14.37,18.75)<br>*16.36 (13.77,18.03)*	16.59 (14.04,18.17)<br>*15.94 (13.47,17.49)*	15.64 (13.16,17.24)<br>*15.02 (12.64,16.62)*	14.99 (12.54,16.65)<br>*14.4 (12.04,16.05)*
	3	16.94 (14.13,18.87)<br>*16.21 (13.51,18.09)*	16.99 (14.18,18.89)<br>*16.25 (13.55,18.12)*	17.02 (14.22,18.88)<br>*16.3 (13.61,18.13)*	17.02 (14.26,18.79)<br>*16.3 (13.66,18.04)*	16.85 (14.22,18.51)<br>*16.17 (13.62,17.79)*	16.38 (13.84,17.97)<br>*15.74 (13.28,17.3)*	15.51 (13.04,17.12)<br>*14.9 (12.53,16.5)*	14.99 (12.54,16.65)<br>*14.4 (12.04,16.05)*
	6	16.82 (14.05,18.69)<br>*16.1 (13.45,17.93)*	16.84 (14.08,18.7)<br>*16.12 (13.46,17.93)*	16.85 (14.12,18.65)<br>*16.14 (13.49,17.9)*	16.81 (14.12,18.53)<br>*16.11 (13.51,17.8)*	16.61 (14.01,18.25)<br>*15.94 (13.42,17.56)*	16.14 (13.62,17.74)<br>*15.5 (13.06,17.09)*	15.37 (12.9,16.99)<br>*14.76 (12.39,16.36)*	14.99 (12.54,16.66)<br>*14.4 (12.04,16.05)*
	12	16.64 (13.88,18.49)<br>*15.94 (13.33,17.76)*	16.64 (13.89,18.47)<br>*15.94 (13.34,17.72)*	16.62 (13.89,18.39)<br>*15.92 (13.33,17.64)*	16.52 (13.86,18.23)<br>*15.83 (13.29,17.51)*	16.29 (13.72,17.93)<br>*15.62 (13.14,17.25)*	15.82 (13.32,17.44)<br>*15.19 (12.78,16.78)*	15.19 (12.74,16.83)<br>*14.59 (12.22,16.2)*	14.99 (12.54,16.66)<br>*14.4 (12.04,16.05)*
	18	16.53 (13.8,18.39)<br>*15.84 (13.23,17.65)*	16.52 (13.8,18.35)<br>*15.83 (13.24,17.61)*	16.47 (13.77,18.26)<br>*15.79 (13.23,17.53)*	16.35 (13.71,18.06)<br>*15.67 (13.16,17.35)*	16.1 (13.55,17.74)<br>*15.44 (13,17.05)*	15.64 (13.17,17.26)<br>*15.01 (12.62,16.62)*	15.11 (12.67,16.76)<br>*14.52 (12.14,16.14)*	14.99 (12.54,16.66)<br>*14.4 (12.04,16.05)*
	24	16.47 (13.71,18.35)<br>*15.78 (13.15,17.62)*	16.44 (13.7,18.29)<br>*15.75 (13.15,17.57)*	16.38 (13.68,18.19)<br>*15.71 (13.11,17.46)*	16.24 (13.61,17.98)<br>*15.58 (13.04,17.25)*	15.99 (13.46,17.62)<br>*15.34 (12.89,16.96)*	15.56 (13.08,17.19)<br>*14.93 (12.56,16.52)*	15.08 (12.64,16.73)<br>*14.49 (12.12,16.11)*	14.99 (12.54,16.66)<br>*14.4 (12.04,16.05)*
	36	16.4 (13.64,18.29)<br>*15.71 (13.07,17.56)*	16.36 (13.63,18.23)<br>*15.68 (13.05,17.49)*	16.3 (13.6,18.11)<br>*15.63 (13.02,17.39)*	16.14 (13.51,17.91)<br>*15.49 (12.95,17.2)*	15.89 (13.33,17.54)<br>*15.25 (12.79,16.87)*	15.49 (13.02,17.13)<br>*14.86 (12.49,16.46)*	15.08 (12.63,16.73)<br>*14.48 (12.11,16.11)*	14.99 (12.54,16.66)<br>*14.4 (12.04,16.05)*
	48	16.36 (13.6,18.29)<br>*15.69 (13.04,17.56)*	16.33 (13.58,18.23)<br>*15.65 (13.03,17.49)*	16.27 (13.55,18.1)<br>*15.6 (12.99,17.37)*	16.11 (13.48,17.88)<br>*15.45 (12.9,17.17)*	15.86 (13.29,17.52)<br>*15.22 (12.75,16.85)*	15.48 (13,17.12)<br>*14.85 (12.47,16.45)*	15.09 (12.64,16.74)<br>*14.49 (12.12,16.11)*	14.99 (12.54,16.66)<br>*14.41 (12.04,16.05)*
	60	16.35 (13.6,18.28)<br>*15.68 (13.03,17.55)*	16.32 (13.58,18.22)<br>*15.64 (13.02,17.48)*	16.25 (13.54,18.1)<br>*15.59 (12.98,17.36)*	16.1 (13.45,17.87)<br>*15.44 (12.9,17.17)*	15.85 (13.28,17.52)<br>*15.21 (12.74,16.85)*	15.48 (12.99,17.12)<br>*14.85 (12.47,16.45)*	15.08 (12.63,16.74)<br>*14.49 (12.12,16.11)*	14.99 (12.54,16.66)<br>*14.41 (12.04,16.05)*
